# Autonomous robotic operation controlled by wave-based neuromorphic hardware

**DOI:** 10.1038/s41467-026-77661-3

**Published:** 2026-09-10

**Authors:** Jacob Zohar, Daniele Pinna, Gerrit van der Laan, Thorsten Hesjedal, C. K. Safeer

**Affiliations:** 1 Clarendon Laboratory, Department of Physics, University of Oxford, Oxford, UK; 2 Diamond Light Source, Harwell Science and Innovation Campus, Didcot, UK

**Keywords:** Electronic and spintronic devices, Information theory and computation

## Abstract

Equipping robots with a physical, brain-inspired computing unit is a key step towards achieving true autonomy in intelligent machines; however, the practical realization of such neuromorphic hardware remains a major challenge. Because both software- and hardware-based spiking neural network approaches demand substantial training and energy consumption, an architecturally simple neuromorphic platform is required to enable scalable and energy-efficient robotics. Here, we experimentally demonstrate robotic control driven by a neuromorphic hardware based on physical wave interactions. Using a water-based experimental system that provides direct visual access to wave-network dynamics, we establish a wave-based reservoir computing framework that achieves near-perfect accuracy in robotic vehicle obstacle recognition, followed by real-time autonomous motion with obstacle avoidance controlled entirely by this wave-based hardware. Through micromagnetic simulations, we extend this concept to electrically excited spin waves in a magnetic nanodevice operating at gigahertz frequencies, outlining an approach toward solid-state robotic chips based on wave computing. These results position wave-based computation as a scalable and energy-efficient alternative to conventional digital neuromorphic architectures for next-generation intelligent machines.

## Abstract (web summary)

The authors demonstrate autonomous robotic control using wave-based neuromorphic hardware. Physical wave interference performs reservoir computing for obstacle recognition and real-time robot navigation, with a route toward solid-state implementations based on spin waves.

## Introduction

As AI workloads demand increasing levels of computational efficiency and parallelism, CMOS-based hardware will need to be complemented, and in some cases replaced, by neuromorphic architectures^1–3^. This transition is especially critical in robotics^4–7^, where current reliance on deep neural network (DNN)-based approaches faces major limitations. Firstly, extensive training on large datasets using power-hungry GPU clusters^2^ is at odds with the low-power computing budgets of many autonomous systems. The reliance on cloud-based connectivity to these data centres is impractical for real-time operation in remote or adversarial environments^8^, and also raises privacy and security concerns. Furthermore, pre-trained models often fail to adapt to unfamiliar and changing real-world scenarios^9^. Together, these pose challenges for various robotic tasks.

Real-time reactive control is a core requirement across many dynamic robotic systems, especially in autonomous vehicles. From warehouse automation systems to aerial drone, robots must operate across multiple timescales with high precision, often in complex, unpredictable environments such as underwater terrains, disaster zones, or planetary surfaces^8^. Conventional DNN-based control of robotic vehicle typically uses batch processing, in which input data is collected and processed periodically during its motion. This leads to redundant, energy-inefficient computation in static environments. In contrast, biological brains are event-driven, initiating processing only in response to dynamic changes in sensory input^10,11^. Emulating this strategy in robotics promises substantial gains in efficiency and responsiveness. Achieving it requires two key components: (i) low-power neuromorphic hardware and (ii) sensory systems that trigger computation only upon dynamic changes in the environment. In this work, we introduce a robotic hardware platform that meets both criteria.

A variety of physical systems have recently been proposed for neuromorphic computing hardware^12^, including CMOS-based solutions^13,14^, memristors^15–17^, spintronic nano-oscillators^18^, superconducting circuits^19^, and photonic systems^20,21^, demonstrating tasks such as image and speech recognition. However, crossbar-style, weight-programmed architectures have major shortcomings: the need for three-dimensional nanofabrication and integration, device non-uniformity, and energy-intensive weight tuning with substantial peripheral circuitry^22^, limiting their suitability for low-power adaptive robots. An alternative is to pursue neuromorphic systems that are sufficiently complex for robotic tasks yet architecturally simple. One guiding idea is a collective brain dynamics approach^23^, where computation arises from the collective activity of neural populations rather than individual units. A closely related framework is reservoir computing (RC), which harnesses the inherent nonlinear dynamics of a single system, the reservoir^24–26^. In RC, only the output layers are trained (see Supplementary Information S1), significantly reducing the computing requirements and energy consumption. The hardware implementation is also simpler, as it primarily relies on the fabrication of a single physical system and is robust to device variability^26^. Yet, realizing such a reservoir that is electrically controllable and chip-integrable remains an open challenge^12^.

**Fig. 1:**
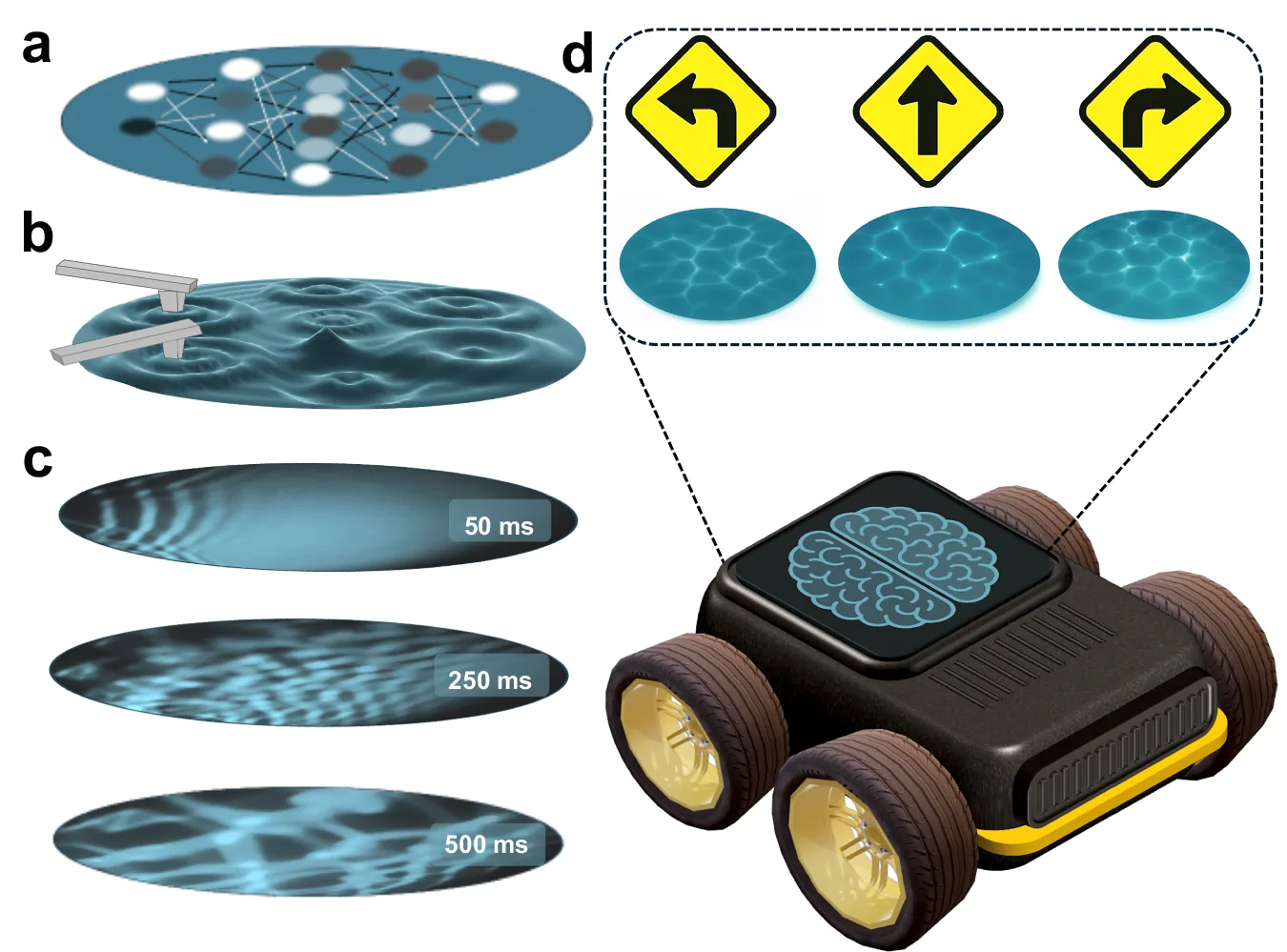
Conventional neural network versus wave-based computing for autonomous robotic vehicle motion. **a** A simple schematic of conventional neural network composed of neurons (circles) with different activation levels and synaptic connections (arrows). Such networks typically contain millions of neurons and connections and evolve nonlinearly by iteratively adjusting synaptic weights during training, which demands excessive computing resources. **b** A 3D illustration of a wave system in which constructive and destructive interference patterns emerge. Once initiated by the input actuators, the wave network itself evolves into a high-dimensional state without external weight tuning, consuming no additional energy. **c** Experimental 2D images corresponding to the wave-evolution illustration shown in (**b**), obtained in our water-wave experiments. Bright and dark contrasts correspond to constructive and destructive interference, respectively. The patterns are captured at 50, 250, and 500 ms after input excitation. The colours have been adjusted for better visibility. **d** Schematic of the robotic control principle demonstrated in this work. The robotic car is controlled by neuromorphic hardware based on wave computing. During its motion, the robot senses obstacles and generates input waves. The output interference patterns that correspond to a class of obstacle configurations share distinct features, which the robot learns during training. This allows the robot to make decisions and take the required action based on the wave patterns, e.g., moving forward or turning right/left (arrows).

Wave systems with nonlinear dynamics are promising candidates for RC^27^. Unlike conventional neural networks that require explicit tuning of millions of neurons and synapses (Fig. 1a), waves self-evolve and interfere, naturally providing both the connectivity and transformations required by RC (Fig. 1b). Because different wave media operate across broad spatial and temporal scales, RC devices can be tailored to handle tasks of varying complexity and speed. This versatility is promising for building hardware composed of interconnected reservoirs capable of real-time, parallel processing of inputs with diverse spatial and temporal characteristics, an approach particularly advantageous for AI applications requiring processing of multiple sensory data streams simultaneously, such as multitasking robots^4,7^. Despite these advantages and multiple theoretical studies supporting their utility^27–29^, along with a few experimental studies in specialized metamaterials or specific geometries^30–32^, an experimental demonstration of a universal framework for wave-interference-based neuromorphic computing remains elusive. Additionally, wave-based neuromorphic computing hardware for robotics has not yet been demonstrated, either theoretically or experimentally.

In this work, we first experimentally demonstrate wave-based neuromorphic computing for a robotic obstacle-avoidance task. We then demonstrate real-time robotic operation controlled entirely by wave-based neuromorphic hardware (Fig. 1d). Finally, using micromagnetic simulations, we show that nanoscale spin waves operating at nanosecond timescales can be used to solve the same robotic tasks.

## Results

### Demonstration of wave-based reservoir computing

**Fig. 2:**
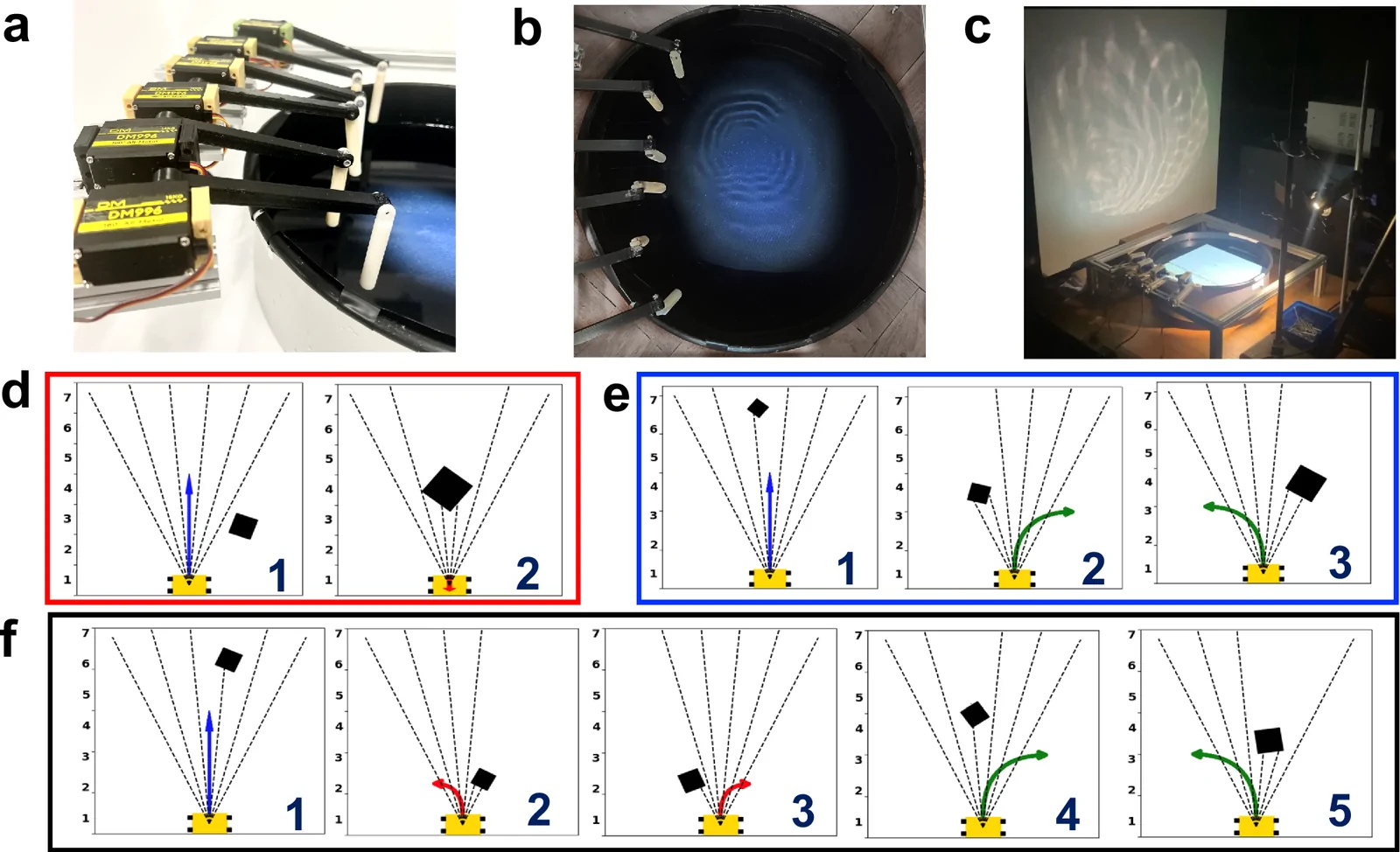
Water-wave-based computing setup and visualization of the dataset. **a** Input wave generators: 3D-printed shafts attached to servo motors periodically dip into the water surface to create controlled excitations. **b** Circular, water-filled metal container used as the reservoir. **c** Illumination and readout: an LED torch mounted at ~45° relative to the water surface projects the surface reflections onto a screen, and a real-time camera facing the screen records the evolving interference patterns for analysis. **d** 2-output task: Class 2 corresponds to an obstacle lying within the near field (minimum-distance range of 4), while Class 1 corresponds to either no obstacle being present within the sensor range or the obstacle being beyond the minimum-distance range. **e** 3-output task: Class 1 corresponds to either no object being present or the object being farther than a threshold distance (range 4). In the remaining two classes, an obstacle is detected within the threshold distance and is classified as being on either the left or the right side. **f** 5-output task: the first class corresponds to either no object being present or the object being beyond the far-distance threshold (range 4). The remaining classes correspond to obstacles grouped by distance, from “close” (range 2) to “far” (range 4), and then by lateral position (left or right). In all panels, the robot (yellow) is positioned along the lower boundary. Dashed rays indicate the six distance-sensor directions, the y-axis represents distance values ranging from 1 to 7, the black polygon marks the obstacle, and coloured arrows trace the resulting robot path to be taken for each class according to the obstacle position.

**Fig. 3:**
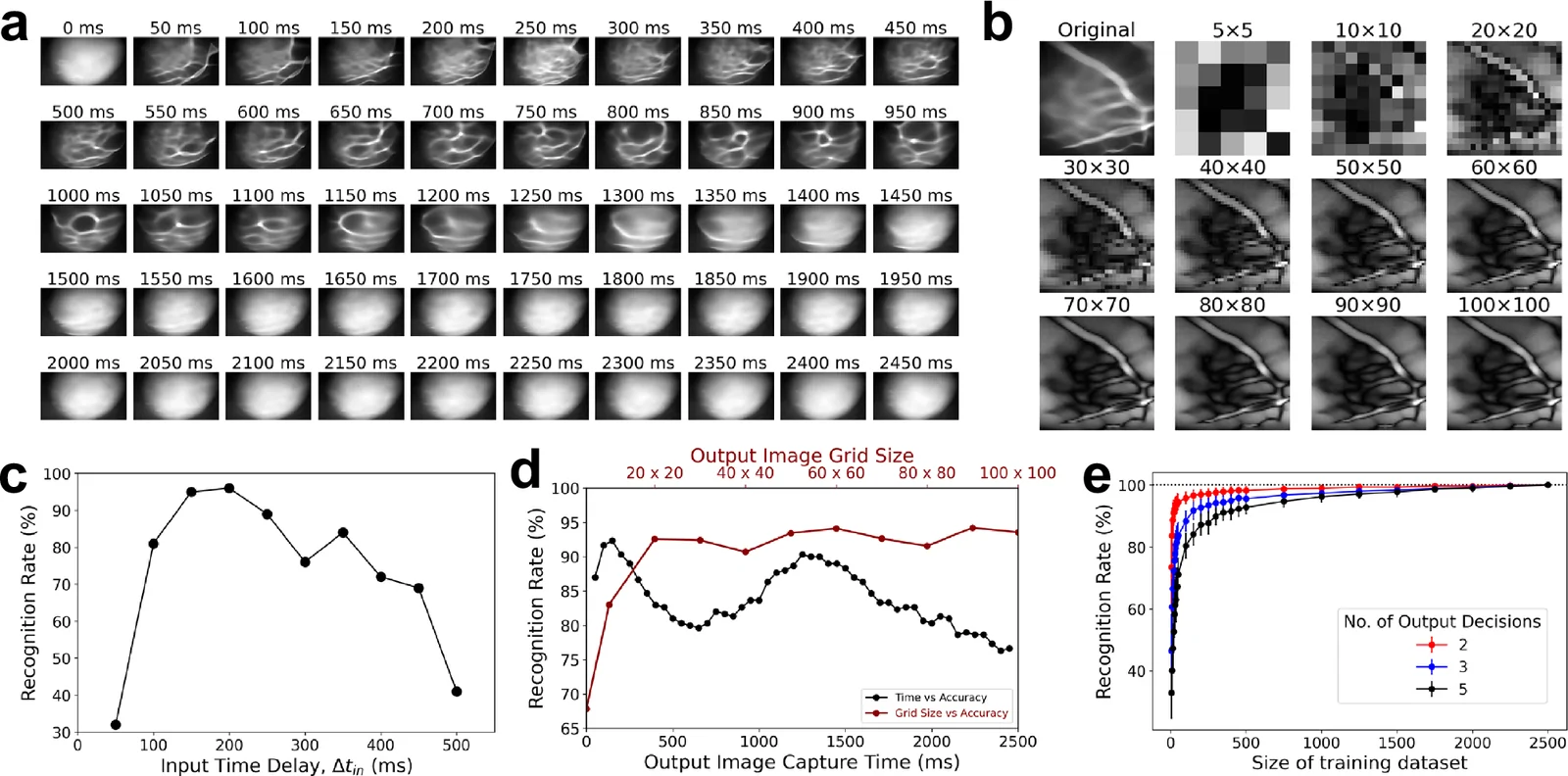
Input encoding, output readout optimization, and RC performance. **a** An example sequence of 50 output images captured every 50 ms following input-wave excitations. The first frame (top) was taken before input excitation and serves as a static reference. **b** Example output images down-sampled into grids of varying resolutions (5 × 5 to 100 × 100), derived from the original output images in the first column. **c** Input-encoding optimization: recognition rate for the 5-output task as a function of the input time delay Δ*t*_in_ using 250 training and 100 test samples. The accuracy peaks at 200 ms, which balances excessive input overlap at short delays against weak interaction at long delays. **d** Output-readout optimization: recognition rate versus the time at which the output interference image is captured (black curve) and versus the spatial grid used to sample the output image (red curve). The recognition rate peaks at  time ~ 150 ms and the performance saturates above grid size ~ 20 × 20. Both curves are averages over the 2-, 3-, and 5-output tasks with 250 training and 100 test samples (see Supplementary Information S3). The example images used for training are shown in (**a**, **b**). **e** Learning curves: obstacle recognition rate with error bars for 100 test samples versus the number of training samples for the 2-, 3-, and 5-output tasks; with Δ*t*_in_ = 200 ms and using a single output frame down-sampled to a 50 × 50 grid captured at 150 ms after input excitation. Accuracies reach up to 100% for all tasks. The number of training sets required to reach maximum accuracy increased with task complexity (5-output > 3-output > 2-output).

When experimentally implementing RC for AI tasks, the reservoir effectively acts as a black box, making it difficult to determine how computational performance relates to the richness and complexity of the reservoir’s internal dynamics. This has been a major challenge in developing wave-based RC, as an experimental platform that enables direct control, visualization, and analysis of a wave medium with complex and evolving dynamics has been lacking. To overcome this, we built an experimental system based on water waves. Water-based setups have previously served as experimental model systems in physics and computing, including demonstrations of logic devices^33^, solutions to XOR problems^34^, and studies of topological and quantum-like wave phenomena^35,36^. Similarly, we built a table-top water-wave-based RC experimental setup (Fig. 2a–c and see “Methods”) comprising three parts: the input, reservoir, and output. A circular metal container filled with water forms the reservoir (Fig. 2b). Six actuators periodically dip into the water surface to generate waves following a defined timing sequence (Fig. 2c). The water surface is illuminated by an LED torch, and the reflected caustic pattern is projected onto a screen and recorded by a camera (Fig. 2c), as output images (Figs. 1c and 3a), allowing direct analysis of the real-time evolution of wave dynamics and their potential for RC. This experimental setup has a configuration similar to the previously reported water-wave RC system^34^; however, it incorporates several advanced features, including an Arduino-based electronic circuit for temporally controlled and reproducible input generation on the ms timescale, a compact design that ensures strong wave interference, and placement on a vibration-isolated table to minimize external perturbations (see “Methods” and Supplementary Information S2).

The experimental water-wave RC system was then employed to test its suitability for a robotic motion task involving obstacle avoidance. This task is typically achieved by training the robot to assess its environment and manoeuvre accordingly^37^. For this, we developed a custom script to generate and visualize the training and testing datasets (Fig. 2d–f). The robot sensed its environment using six forward-facing distance sensors, each returning a discretized distance value from one to seven based on obstacle position, thereby forming a six-element input vector. Because obstacles could appear in random positions, the combination of sensor readings yielded 7⁶ = 117,649 possible input scenes, providing sufficiently large datasets for experimentation. We defined three classification tasks with 2, 3, and 5 output classes, respectively, requiring the robot to categorize random input scenarios to make corresponding manoeuvre decisions. Three tasks with progressively increasing numbers of output classes were designed to evaluate RC performance under increasing levels of decision complexity. In the 2-output case, the robot classified input scenes based on whether an obstacle was within a minimum distance, deciding either to stop or move forward (Fig. 2d). In the 3-output case, the object’s position, i.e., left, right, or no object, was determined and the robot turned accordingly (Fig. 2e). In the 5-output case, proximity (close/far) and side (left/right) were jointly classified, and the robot executed sharp turns for ‘close’ and smoother turns for ‘far’ and proceeded straight ahead if no obstacle was detected (Fig. 2f).

Experimentally, the inputs were time-encoded, i.e., the six actuators generated water waves according to the distances measured by the six sensor lines, with each of the seven possible distance levels mapped to a distinct wave initiation time. The resulting waves propagated, interfered, and evolved dynamically, producing unique output interference patterns that were captured and used for training and testing with a support vector machine (SVM) (see “Methods” and Supplementary Information S1). The discrete timing map should be viewed as an input transduction step rather than the computation itself. The temporally evolving, spatially extended wave medium, in which propagation, interference, and dispersion occur, mixes the encoded inputs into a high-dimensional spatiotemporal state and thus acts as the reservoir. We first optimized the input encoding procedure, followed by optimization of the output training, and finally evaluated the obstacle recognition accuracy.

According to RC theory, the classification accuracy is maximized when the input encoding timescale matches the reservoir’s intrinsic dynamics^26^. We first experimentally demonstrated this concept as follows: with the input distance values *d* = {1, 2, 3, 4, 5, 6, 7} mapped to input wave initiation times *t*_*in*_ = {50, 100, 150, 200, 250, 300, 350} ms, i.e., with a fixed delay of 50 ms between adjacent inputs (Δ*t*_*in*_ = 50 ms), the 5-output task was studied using 250 training and 100 test samples. After all inputs were excited, 25 interference images were recorded over 1.25 s for training and testing. The experiment was then repeated for different Δ*t*_*in*_ values from 50 to 500 ms. The accuracy peaked at Δ*t*_*in*_ = 200 ms (Fig. 3c). For short delays (e.g., 50 ms), the inputs were initiated almost simultaneously, producing similar interference patterns irrespective of the input values. For long delays (e.g., 400 ms), reduced temporal overlap weakened the input correlations. The intermediate setting of 200 ms achieved a balance between separability and interaction, yielding the highest accuracy.

Next, we optimized the minimal temporal and spatial information required for output training. Following input-wave excitation, the interference patterns evolved dynamically before eventually damping out. A sequence of output image frames was captured at the centre of the water reservoir during this evolution (Fig. 3a), providing temporal and spatial information of the reservoir’s output state. Although all captured frames could be used for training, this approach is computationally and energy intensive, and includes processing of significant redundant information. Instead, RC relies on the idea that maximum classification accuracy can be achieved by training on a single reservoir output state corresponding to the time point with the highest degree of information mixing. To determine this point in time, we captured output images every 50 ms over a 2.5-s interval following input-wave excitation, as shown in the example in Fig. 3a. Using these images, we evaluated obstacle-recognition rates for the 2-, 3-, and 5-output classification tasks as a function of capture time (see Supplementary Information S3 and Supplementary Fig. 3a). The overall average of the three tasks is shown in Fig. 3d (black plot), where the accuracy peaked at 150 ms, corresponding to the moment when the reservoir output state contained the highest degree of wave information mixing. We have also observed a second peak near 1400 ms, which is attributed to the mixing of reflected waves and serves as a signature of short-term memory in the system. This is promising for extending wave computing to many AI applications that require temporal data processing (see Supplementary Information S3 for a detailed discussion).

Using the optimized input encoding interval Δ*t*_*in*_ = 200 ms and training on a single output image captured at 150 ms, down-sampled to a 50 × 50 grid, we then evaluated the accuracy of 2-, 3-, and 5-output classification tasks for 100 test samples as a function of the number of training samples. The resulting plot is shown in Fig. 3e, where recognition rates reached up to 100% for all three tasks. As expected, accuracy increased with training-set size and saturated at relatively modest data volumes. Higher task complexity (5 versus 3 or 2 outputs) required larger training sets, consistent with the expectation that greater decision complexity demands more information for effective learning. These results demonstrate that classification accuracy up to 100% can be achieved from a single, time-selected output frame, establishing a low-energy, ultrafast, wave-based RC approach with minimal training requirements.

We further compared classification performance using the wave-based RC with that of a linear SVM without wave processing (Supplementary Information S1 and Supplementary Fig. 1). The wave-based system clearly outperformed the SVM, particularly in the more complex 5-output classification task. This also confirms that the robotic obstacle-recognition tasks employed in this study are not linearly solvable and demonstrates that the wave mixing of the input information acts as a nonlinear kernel that transforms the input data into a suitable representation where linear separation becomes possible, an essential requirement for practical RC.

### Wave-mediated robotic motion with obstacle avoidance

**Fig. 4:**
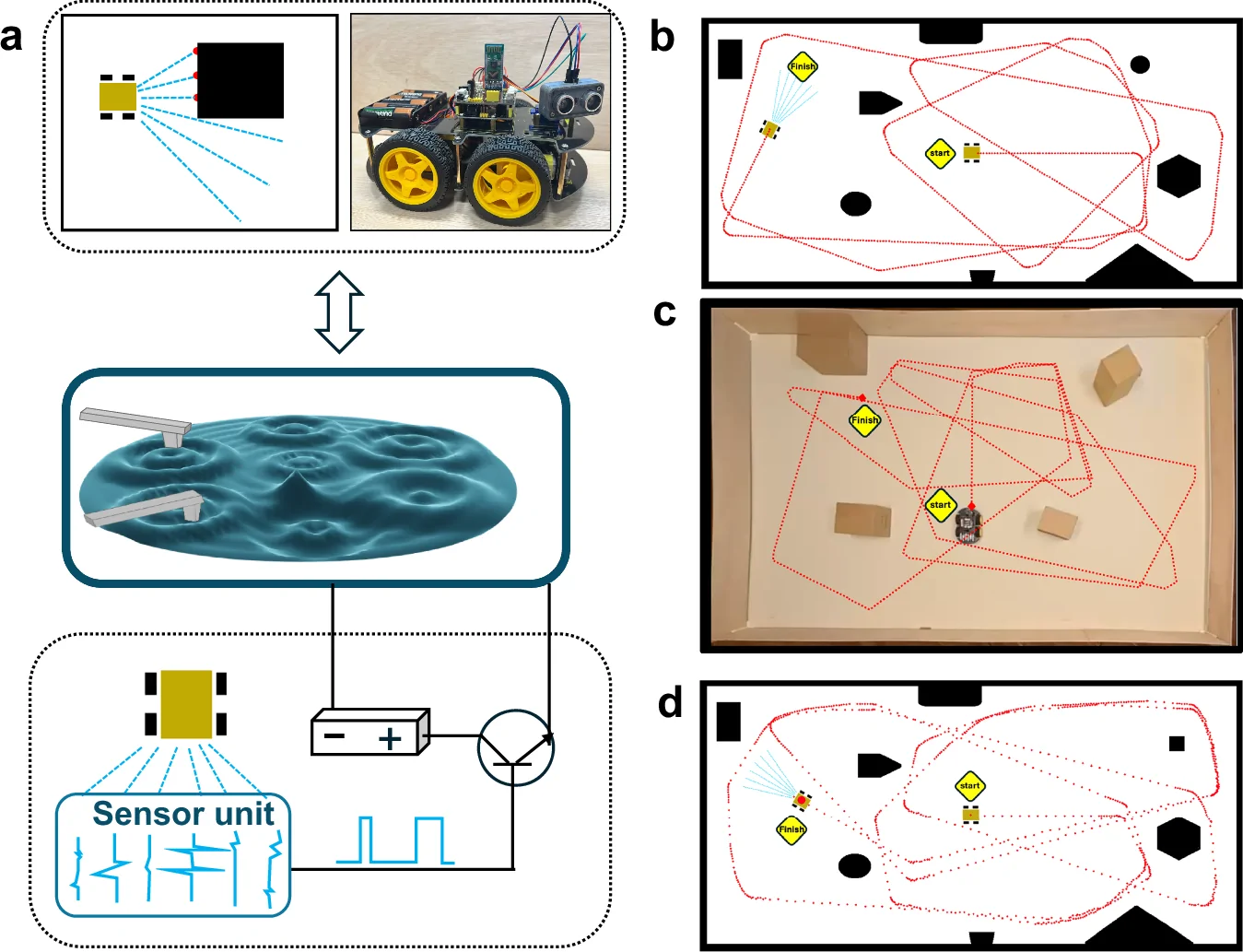
Wave-based robotic motion with obstacle avoidance in simulation and experiment. **a** Top panel: schematic of the virtual robotic vehicle equipped with six obstacle-sensing rays (dashed) at different angles, and a photograph of the Arduino-based vehicle with a rotatable ultrasonic distance sensor and Bluetooth module. Both operate by wirelessly communicating with the water-wave-based neuromorphic hardware (shown in the middle panel). Bottom panel: conceptual circuit for event-driven control in the simulated robot motion. All input actuators of the water-wave reservoir are powered from a common +5 V power supply, and a transistor switch is placed between the supply and the computing unit to turn the unit on or off. The measured distance is first analysed by a sensor unit whose trigger output is connected to the transistor gate. The transistor switch is activated only when there is a change in the input sensory signals, enabling event-driven robotic operation. **b** Trajectory of the virtual robot (red dotted line) extracted from Supplementary Video 1. The robot moves in a bounded arena containing static obstacles (black), transmitting distance data to the water-wave computing unit and receiving manoeuvre commands in return. **c** Trajectory from the physical-robot experiment, extracted from Supplementary Video 2, showing closed-loop motion controlled by the wave-based computing unit; the robot moves within a rectangular arena, avoiding wooden boundaries and randomly placed obstacles. **d** Trajectory of the virtual robot motion using event-based control in (**a**), extracted from Supplementary Video 3. The circle on the car indicates the transistor state (red/green for transistor off/on; see Supplementary Video 3). The dotted trace illustrates the path: larger dot spacing indicates faster motion between events, while tighter spacing reflects slowdowns near obstacles when computation is invoked.

Next, real-time robotic motion controlled by wave computation (Fig. 1d) was performed. For simplicity, the 2-output decision model was used (object within minimum range vs. no object), first with a robotic car in a virtual environment and then with a physical car (Fig. 4a, top panel). The virtual robotic car, implemented in Pygame (see “Methods”), carried six distance sensors and moved in a rectangular arena with randomly placed obstacles (Fig. 4b). The physical robotic car, equipped with an ultrasonic distance sensor and a Bluetooth module for bidirectional communication with the water-wave system (see “Methods”), moved in a 3 m × 2 m obstacle course (Fig. 4c). For both cases, the control loop operated as follows: the car measured distances, sent the discretized values to the water-wave reservoir where the time-encoded input actuators generated the corresponding waves, the resulting interference pattern was imaged in real time and classified by the trained model, and the movement command was then sent back to the robotic car. If no obstacle was predicted, the robot moved forward; otherwise, it executed a ~30° turn to avoid the obstacle before proceeding. In a conventional batch processing mode, the car periodically stopped to measure distances, transmitted the data to the wave-based system at fixed intervals, and then executed the returned command as shown in Supplementary Video 1 (virtual car) and Video 2 (real car). The resulting trajectories are summarized in Fig. 4b, c, where dotted lines trace the robotic motion path. Across these runs, the car followed collision-free trajectories, confirming a real-world demonstration of autonomous robotic motion with obstacle avoidance controlled by wave-based neuromorphic hardware.

During its motion using the batch processing mode, the robot often encountered scenarios with no obstacles, leading to redundant computations of identical inputs. To mitigate this, we adopted an event-based control strategy in which neuromorphic processing is triggered only by significant changes in sensory input. In hardware, one method that this can be implemented is by converting distance-sensor signals to a baseline voltage that gates the wave-computing unit via a transistor switch (a proposed circuit is shown in Fig. 4a, bottom panel). However, to integrate the transistor switch practically, both the sensory unit and the RC system need to be integrated onto a chip mounted on the robotic car. In our current setup, the water-wave RC module is off-board and communicates wirelessly, making direct integration of the transistor switch not feasible. We therefore implemented the event-based logic in the virtual robotic car (see Supplementary Video 3 and Fig. 4d). Here, we placed a virtual transistor switch between the sensor signal transmission unit and the RC system, indicated by a light turning red and green in Supplementary Video 3. In this mode, when there is no change in the sensory signal, the switch between the power supply and computing unit remains off, and the robot moves continuously without performing RC. Above-threshold changes in the sensory signal send a voltage to the transistor gate, turning on the transistor and thereby activating the RC system, and prompting the robot to make decisions according to the RC. The event mode of operation (Supplementary Video 3) reduced computational duty cycle, resulting in faster overall operation and lower power consumption compared with the batch-processing mode (Supplementary Video 2). Moreover, computation is triggered only when an event occurs, demonstrating the adaptability of event-based robotic operation to changes in the external environment.

### Spin-wave-mediated robotic obstacle recognition

While the water-wave-based experiments established wave-based RC and enabled real-time demonstrations of robotic motion, practical on-chip robotic systems require electrically addressable, nanoscale wave media operating at ultrafast timescales within solid-state devices. Spin waves (SWs)^38^ satisfy these criteria: they are electrically excitable, can exhibit strong intrinsic nonlinearity, possess nanometre-scale wavelengths, and operate at GHz frequencies^39^ (see Supplementary Information S4). We therefore investigated SWs as a promising future chip-integrable platform for wave-based neuromorphic control.

**Fig. 5:**
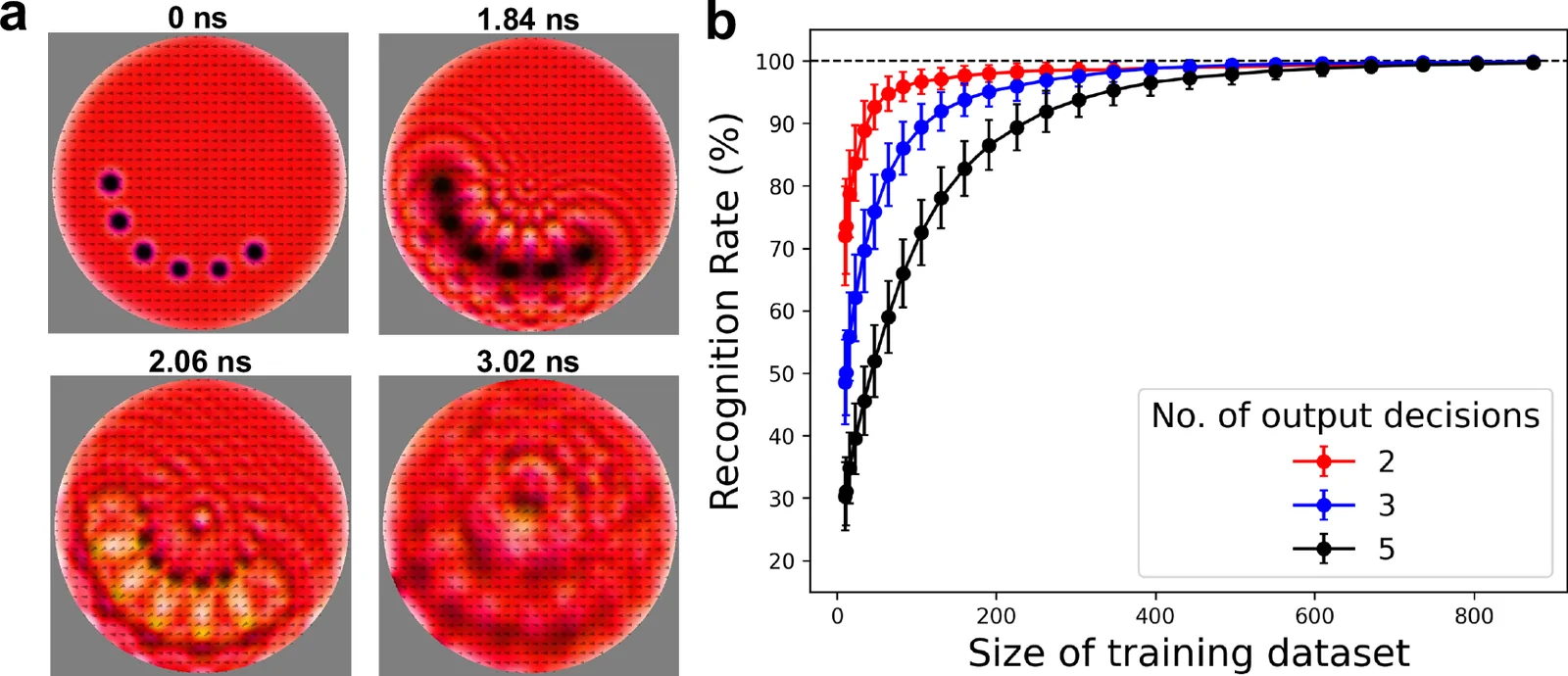
Micromagnetic simulation of the spin-wave-based reservoir and recognition rates. **a** Time-evolution snapshots of the out-of-plane magnetization showing interference of spin waves excited from six input regions (black circles) via VCMA (at 0, 1.84, 2.06, and 3.02 ns). **b** Recognition accuracy versus number of training samples for the 2-, 3-, and 5-output classification tasks (red, blue, and black). Accuracy increases with training-set size and saturates after a few hundred examples.

Electrical excitation of SWs from local electrodes, using either current-induced spin transfer torques or variations in voltage-controlled magnetic anisotropy (VCMA), has already been experimentally demonstrated^40–42^. While interference and readout of multiple nanoscale spin waves^43^ remain active experimental challenges, their dynamics can be quantified with micromagnetic simulations^44,45^ (see Supplementary Information S5 for simulation details). The spin-wave reservoir consists of a circular ferromagnetic CoFeB thin film with a diameter of 1 μm and a thickness of 1.5 nm, with a saturation magnetization (*M*_*sat*_) = 1MA/m and an exchange stiffness (*A*_*ex*_) = 15 pJ/m. Using VCMA-driven excitation at six circular input transduction regions with a diameter of 50 nm, we simulated^45^ propagation and interference of SWs on nanosecond timescales (Fig. 5a) and evaluated 2-, 3-, and 5-output classification tasks with the same training/testing protocol used for water waves. Again, we achieved accuracies up to 100% across all tasks (Fig. 5b and Supplementary Fig. 4). Compared with the water-wave experiment, less training was required to achieve maximum accuracy (Supplementary Fig. 5b), likely due to the richer spatial features of the SW interference (Supplementary Fig. 5a).

A practical SW RC device can follow the simulated geometry: a planar ferromagnetic film embedded with a set of electrically addressable input wave-excitation contacts^46^ and output readout sensors, which can be implemented with standard, CMOS-compatible elements^47^ such as voltage-gated contacts^41^ or magnetic tunnel junctions (MTJs)^43,48^. Crucially, MTJs with dimensions of tens of nanometres have already been demonstrated at industrial scale^47^, providing both electrical access and compact footprints compatible with dense layouts. To confirm the suitability of this device scheme, we repeated the SW simulation classification task using output signals read by an array of MTJs and again achieved maximum accuracy (Supplementary Information S7 and Supplementary Fig. 6). These findings suggest the technical feasibility of a solid-state, GHz spin-wave reservoir as a compact co-processor for robotic control.

For real-time robotic vehicle control, the input from sensory data arrives at a much slower rate (kHz) compared to the operating speed of spin waves (GHz). Even though the system can simply serve as a fast physical co-processor that is interrogated whenever a new sensory update arrives, the fast-processing timescales leave scope for repeated evaluations, time multiplexing, buffering/windowing, and multi-timescale control architectures. Therefore, this approach will be advantageous for future robotic applications requiring complex input-data processing.

## Discussion

We experimentally demonstrated a universal neuromorphic computing approach based on real-time, in situ physical wave interactions, featuring a simplified system architecture compared with conventional digital computing frameworks. We established optimal input encoding and minimal spatiotemporal output training procedures applicable to any RC system. Robotic obstacle recognition with up to 100% accuracy was achieved by training only a single output layer, requiring significantly less power and time than conventional multilayer, backpropagation-based machine-learning models. We further realized real-time autonomous motion with obstacle avoidance driven entirely by wave-based hardware and demonstrated an event-driven mode that minimized redundant computation and energy consumption while maintaining fast, adaptive responsiveness for dynamic tasks. Although our current demonstrations focused on basic robotic obstacle avoidance task, the same framework is readily extensible to more complex behaviours by increasing the input fan-in, combining reservoirs operating at different temporal and spatial scales, and incorporating multiple readout terminals. As a path forward, we extended our studies from macroscopic water waves to nanoscale spin waves, revealing a route toward ultrafast, solid-state, and compact on-chip implementations compatible with standard CMOS fabrication processes. An indicative quantitative benchmarking of the spin-wave RC system against representative neuromorphic and physical RC platforms is provided in Supplementary Sections S8, S9, and Supplementary Table 1, highlighting its advantages in operating speed, energy, training simplicity, device fabrication, and hardware overhead.

More broadly, our approach extends in two key ways. First, the RC principles apply beyond SWs to other wave media, including acoustic (phononic), electromagnetic (radio-frequency), and photonic systems, spanning a range of spatial and temporal scales. Second, the same hardware supports functions beyond the robotic obstacle avoidance task, including event-driven perception and classification, multi-sensory processing, time-series prediction, and anomaly detection at the edge, where low power and real-time operation are critical. Together, these features provide a practical foundation for energy-efficient AI co-processors for embedded devices to autonomous machines.

## Methods

### Water-wave RC experimental setup

The physical reservoir for the water-wave-based RC setup consisted of a round metal container with a diameter of 25 cm and a depth of 5 cm. A frame was built around the container to hold the six servo motors (Fig. 2b). Each servo motor was fitted with a 3D-printed shaft ending in a 4-mm-diameter cylindrical tip (Fig. 2a), which dipped into the water upon actuation to generate water surface waves. The six-input layout was chosen under optical-access constraints rather than by a full geometric optimization. More generally, very small actuator spacing is expected to increase input correlation, whereas very large spacing reduces wave overlap and therefore weakens nonlinear mixing. The best performance should occur in an intermediate regime set jointly by wavelength, damping length, reservoir size, and boundary reflections.

Servo control was achieved through the digital I/O pins of an Arduino board programmed using the Arduino IDE, allowing precise excitation timing. To ensure stable and reproducible excitation of water waves, each servo motor was powered by an external 5 V source, and a stabilization circuit (see Supplementary Fig. 2), consisting of a 1000 µF electrolytic capacitor for each servo, was added to prevent unwanted excitations.

The resulting wave patterns were visualized by positioning an LED at a 45° angle to the water surface, projecting the reflections onto a screen (Fig. 2c). A camera (ELP USB HD camera) capable of real-time image capturing with a speed of up to 15 frames per second, placed opposite to the screen, recorded the evolving interference patterns A Python-based control system synchronized the Arduino and the camera, enabling real-time data acquisition for wave-based computation.

### Wave image processing and classification

The captured water-wave interference images were first processed by subtracting a static reference frame, recorded without wave excitation, to isolate dynamic features from the static background. Each resulting differential image was then down-sampled into a two-dimensional grid. Within each grid cell, the pixel intensity was averaged to generate a compact feature matrix representing the wave pattern.

These matrices were then used for training and testing to calculate the accuracy of classification. For the results shown in Fig. 3e, a dataset of 2500 samples was collected for training. Testing was conducted on 100 samples, while systematically varying the number of training samples from 5 to 2500. This was repeated 50 times with random training/testing datasets, and the classification accuracy was averaged. Such averaging over random data ensured the robustness of our results.

A similar procedure was followed for SW image processing and classification shown in Fig. 5b.

### Integration of virtual robotic car with RC experimental set up

A virtual environment was developed using the Pygame framework to experimentally test robotic motion with obstacle avoidance (Fig. 4a, b). Within this environment, the virtual car stopped at regular time intervals to measure obstacle distances via virtual sensors. These distance values were normalized to a range between one and seven and transmitted to a Python program controlling the water-wave-based RC system. Upon receiving the input, the program activated specific servo motors to generate wave patterns in the water. The resulting interference images were captured and processed, and a trained classification model was used to interpret the wave pattern and predict the presence of obstacles. If no obstacle was detected, the simulated car moved forward. If an obstacle was detected, it executed a ~30° turn to avoid it before resuming motion. Each input-output cycle lasted ~5 s, allowing the water surface to return to equilibrium before the next measurement. To create the videos of the virtual car’s motion shown in Supplementary Videos 1 and 3, frames were saved at regular intervals during motion, and the videos were rendered at 30 frames per second.

### Physical robotic vehicle construction and RC experimental integration

A four-wheeled robotic vehicle (Fig. 4a) was built using an Arduino-based smart car kit equipped with a front-mounted ultrasonic distance sensor (HC-SR04) attached to a servo for angular scanning to measure distances at six angles. The chassis of the Arduino-powered robotic car was built from lightweight ABS plastic. It housed four DC motors connected via a motor driver (L298N module) to the Arduino Uno R3. A 9 V battery pack provided power for the entire system. The HC-SR04 ultrasonic sensor was mounted on a TowerPro-SG90 servo motor, enabling it to scan at six different angles. The Arduino IDE code incorporated logic for distance thresholding and motion control.

Bluetooth communication used a serial protocol. The RX and TX pins of the HC-05 module were connected to the TX and RX pins of the Arduino through a voltage-divider circuit to step down logic levels from 5 to 3.3 V. Sensor data were transmitted via the Bluetooth module to a Python program running on a host computer. The received distance values, normalized to a range between one and seven, served as input to the wave-based RC system. The Python program processed the data, triggered wave generation through servo actuation, and captured the resulting interference patterns. A trained classification model then interpreted the wave pattern to determine the presence of obstacles. Based on the classification result, the program sent movement commands back to the Arduino of the robotic car. This closed-loop system enabled real-time autonomous motion with obstacle avoidance, powered entirely by physical wave dynamics.

The video of robotic motion was captured using a wide-angle camera (DJI Osmo Action 5 Pro). To make the compact video presented in Supplementary Video 2, the speed of the real-time video was increased by a factor of 50.

## Supplementary information

Supplementary Information

Description of Additional Supplementary Files

Supplementary Video 1. Autonomous motion of a virtual robotic car performing obstacle avoidance controlled by wave-based reservoir computing.

Supplementary Video 2. Autonomous motion of an Arduino-based robotic car performing obstacle avoidance controlled by wave-based reservoir computing.

Supplementary Video 3. Event-based operation of the virtual car.

Transparent Peer Review File

## Data Availability

The data supporting the findings of this study are available in the main text and the Supplementary Information.

## Code availability

The code used in this study is publicly available on Zenodo (ref. ^45^) at https://doi.org/10.5281/zenodo.21772974.
