# Supplementary material for: Autonomous robotic operation controlled by wave-based neuromorphic hardware
Source: Nat Commun. 2026 Sep 10;17:10677. doi: 10.1038/s41467-026-77661-3 (PMC13654935; doi:10.1038/s41467-026-77661-3)
Supplement: Supplementary file 1 [file 41467_2026_77661_MOESM1_ESM.pdf]

Supplementary Information

Autonomous robotic operation controlled by wave-based neuromorphic hardware

Jacob Zohar[1], Daniele Pinna[1], Gerrit van der Laan[2], Thorsten Hesjedal[1], C. K. Safeer[1]

[1]Clarendon Laboratory, Department of Physics, University of Oxford, Oxford OX1 3PU, UK

[2]Diamond Light Source, Harwell Science and Innovation Campus, Didcot OX11 0DE, UK

Contents

1. **Support Vector Machines and Reservoir Computing**
2. **Water-Wave-Based Reservoir Computing Setup**
3. **Training on Output Information**
4. **Spin-Wave-Based Reservoir Computing**
5. **Simulation Procedure for Spin-Waves**
6. **Comparison between Spin-Wave and Water Wave RC**
7. **Results Using the MTJ Readout Method for Spin-Waves**
8. **Quantitative Comparison of Spin-Wave RC with State-of-the-Art Neuromorphic Hardware**
9. **Computational benchmarking**
10. **Supplementary Videos Description**

S1. Support Vector Machines and Reservoir Computing

A support vector machine (SVM)[1] is a simple machine learning (ML) algorithm commonly used to carry out classification and regression tasks efficiently. SVMs take a vector of n features (v_1, …, v_n) as their input and compare it to a hyperplane of dimension n-1 to assign it to a class. The goal of the algorithm is therefore to identify the hyperplane that optimally distinguishes inputs into distinct classes, thereby defining the optimal decision boundary.

SVMs are supervised learning algorithms, meaning the training process requires pairs of input vectors matched with their corresponding output classes. As the SVM iterates through the data, it identifies the vectors closest to their class boundary, known as support vectors. The hyperplane is then defined as normal to the axis connecting these support vectors. Since the testing process only involves comparing an input vector to a hyperplane, SVMs provide a computationally efficient method for linear classification, making them the preferred ML algorithm in many situations.

Since SVM classification operates on linear decision boundaries, an accuracy of 100% is attainable only when the data are perfectly linearly separable. To achieve maximum accuracy, the input data must first be transformed using a nonlinear kernel function, which maps the original data into a higher-dimensional vector space where linear separation can be more effectively achieved. Given that different problems will generally require unique transformations with varying nonlinearity, the ideal kernel must provide a universal approach to transformation. RC is an approach to constructing a universal kernel function by employing an interconnected and recurrent system with inherent nonlinear dynamics: the reservoir.

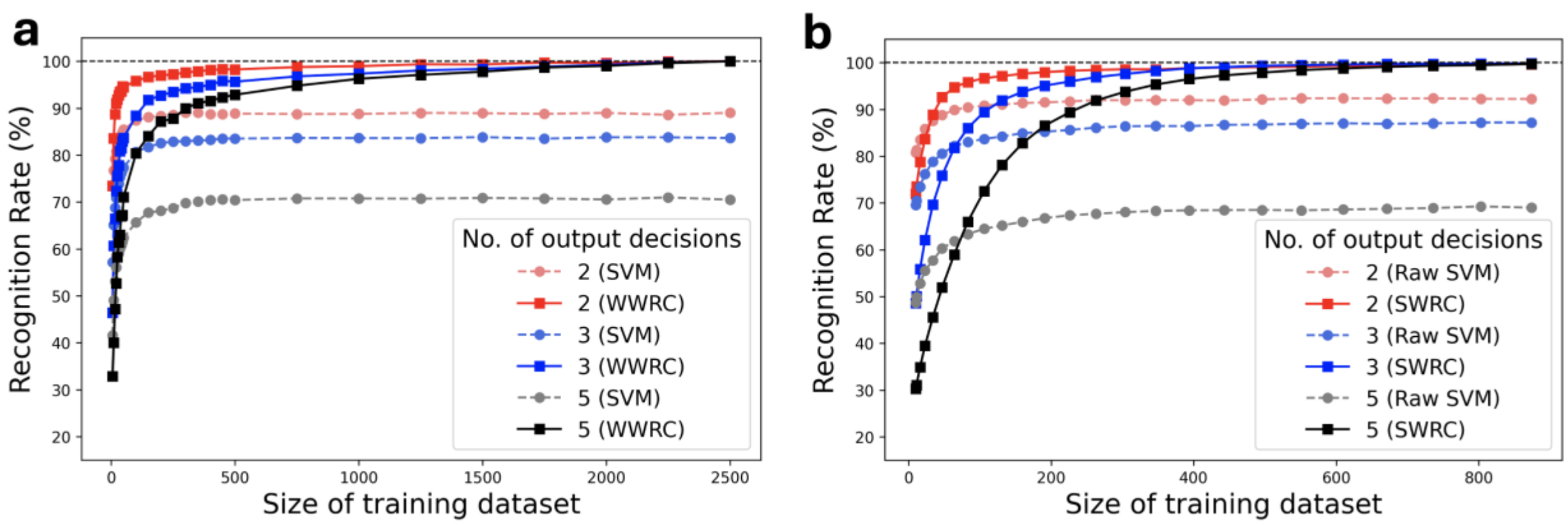

Supplementary Fig. 1. Comparison of the performance of wave-based RC and SVM. *Recognition accuracy as a function of training dataset size for raw SVMs versus using **(a)** water-wave and **(b)** spin-wave reservoir computing (WWRC and SWRC). The curves show the mean across 100 random shuffles of the training data. In all cases, the reservoir computing was able to achieve 100% accuracy, while the raw SVM performance saturates at lower values, demonstrating that the wave-based reservoirs provide the necessary nonlinear transformations of the input data required for linear SVM classification.*

To confirm that our wave reservoir performs nonlinear kernel transformations, we compared the classification of 2-, 3-, and 5-output decision tasks using SVMs with and without passing through the wave reservoir, as shown in Supplementary Fig. 1. For raw SVMs, the accuracy saturated at <100%, indicating that purely linear classification is not possible. Also, maximum accuracy decreased with increasing complexity of the tasks to the 2-, 3-, and 5-output tasks, indicating the need for increasing kernel nonlinearity with increasing complexity of the task. However, for the

wave reservoir, the accuracy of all tasks reached 100%, confirming that it acts as a kernel function enabling perfect classification with a linear SVM.

S2. Water-Wave-Based Reservoir Computing Setup

The physical reservoir for the water-wave-based RC setup consisted of a round metal container with a diameter of 25 cm and a depth of 5 cm. A frame was built around the container to hold the servo motors (see Fig. 2 in the main text). Each servo motor was fitted with a 3D-printed shaft ending in a 4-mm-diameter cylindrical tip, which dipped into the water upon actuation to generate water surface waves.

To ensure stable and reproducible excitation of water waves, the servo motors were driven with high precision using an Arduino-based circuit (Supplementary Fig. 2). Each servo motor was powered by an external 5 V source, and a stabilisation circuit, consisting of a 1000 µF electrolytic capacitor for each servo, was added to prevent unwanted excitations.

The entire setup was placed on a vibration-isolated table within an acoustically insulating enclosure to minimise environmental disturbances such as mechanical vibrations and air currents.

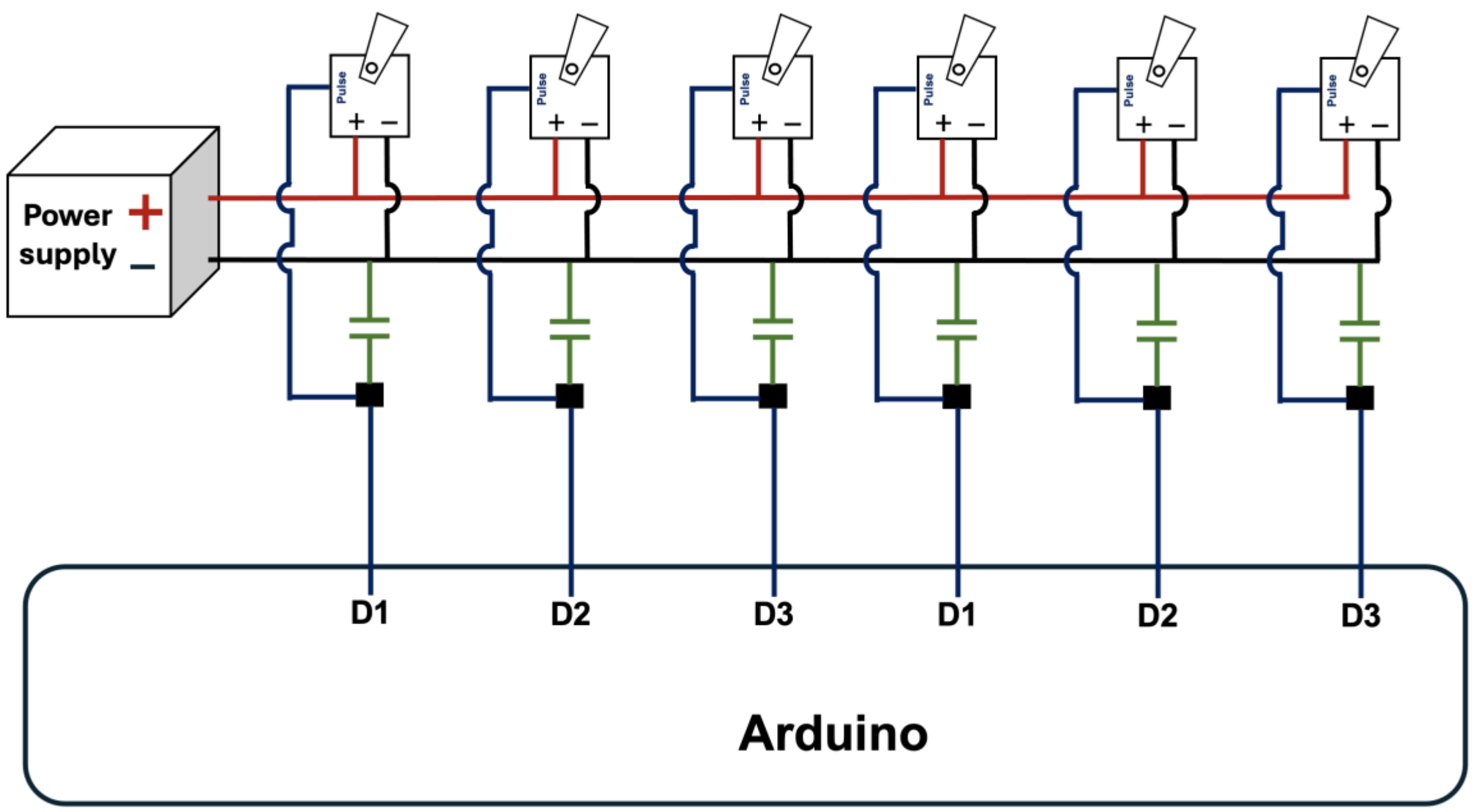

Supplementary Fig. 2: Schematic of the Arduino circuit controlling the servo motors in the water-wave RC system. *Each servo motor is powered by a +5 V supply, and their movements are governed by digital control signals (Arduino pins D1-D6). A 1000 µF capacitor is connected to each input to suppress unwanted current spikes and stabilise motor operation.*

S3. Training on Output Information

To analyze the temporal evolution of the reservoir state, we captured output images every 50 ms over a 2.5-s interval following input-wave excitation as is shown in Fig. 3a in the main text. Initially, we evaluated obstacle-recognition accuracy for the 2-, 3-, and 5-output classification tasks as a function of capture time as shown in Supplementary Fig. 3a. Each curve represents the average of 50 independent training and testing runs, with 250 random training datasets and 100

testing datasets per run. The overall average of the three curves from Supplementary Fig. 3a is shown in Fig. 3d (black plot) in the main text. Along with recognition accuracy peaked at ~150 ms, a secondary peak was observed near 1400 ms, and the comparison with the corresponding wave images (Fig. 3a in the main text) indicates that this arises from reflected waves. These reflections reintroduce distinctive features useful for recognition, suggesting that reflected waves act as a form of short-term memory, merging with new inputs to enable history-dependent pattern recognition. This is an essential capability for tasks such as time-series analysis and speech recognition, and it is promising for extending the wave-computing concepts developed here to other AI applications beyond robotics.

We have also performed the variation of recognition accuracy as a function of grid resolution for 2, 3, and 5 output classification tasks were performed is shown Supplementary Fig. 3b, again evaluated for 100 testing datasets using 250 training datasets, averaged over 50 runs. The overall average of the three tasks is shown in Fig. 3d (red plot) of the main text.

The amount of information required for training depends on the complexity of the task. More complex problems may require higher spatial resolution or additional temporal data, whereas simpler tasks can achieve satisfactory accuracy with less information. For instance, Supplementary Fig. 3c compares the performance of training and testing with a single frame versus 25 frames captured over a 1.25-second interval. As expected, multi-frame training yields better performance, achieving 100% accuracy with fewer training samples due to the inclusion of richer temporal information. For more demanding RC tasks, such extensive training strategies can be adopted as needed.

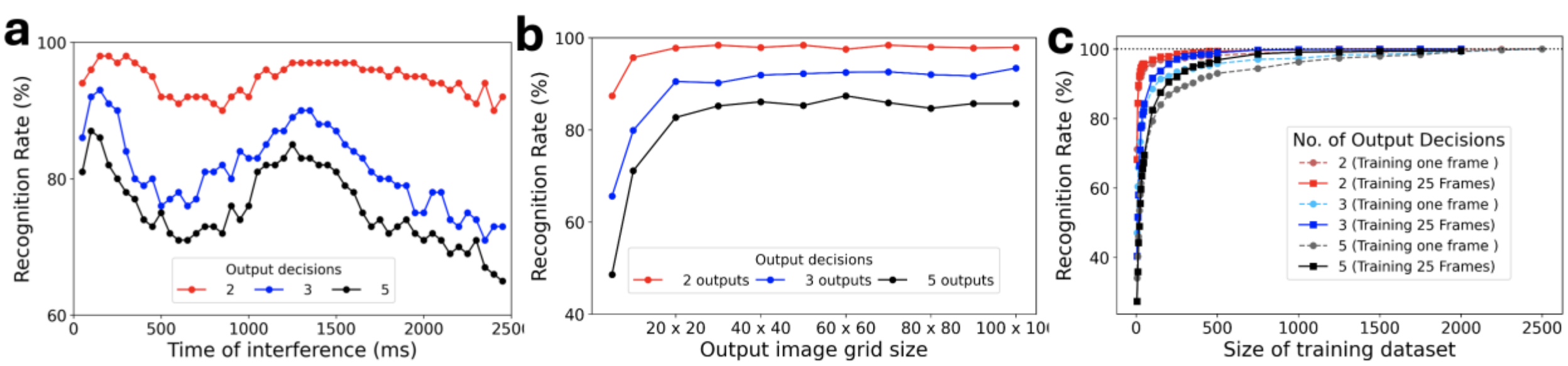

Supplementary Fig. 3. Optimization of output training using water-wave interference images. ***(a)*** *Recognition accuracy as a function of time at which the output interference pattern was captured (an example of sequence of image shown in Fig. 3a of the main text), evaluated for the 2-, 3-, and 5-output classification tasks. Each curve represents the mean over 50 independent iterations. The overall average is shown in Fig. 3d of the main text. The oscillatory behavior of the plot arises from interference with the first reflected waves.* ***(b)*** *Recognition accuracy as a function of output grid resolution (5 × 5 to 100 × 100) for the 2-, 3-, and 5-output classification tasks. Examples shown in Fig. 3b of the main text. Coarsely spaced grids provide less spatial detail and therefore reduce accuracy. Note that the performance saturates beyond ~20 × 20. The overall average is shown in Fig. 3d of the main text.* ***(c)*** *Training efficiency comparison between single-frame and multi-frame output images. The recognition rate as a function of the number of training samples is shown for the 2-, 3-, and 5-output decision-making tasks. The dotted lines represent single-frame training using the optimal time (150 ms) and the solid lines multi-frame training with 25 sequential frames over 1.25 s. Multi-frame training yields higher accuracy with fewer samples, whereas single-frame training requires more data but achieves comparable saturation performance.*

S4. Spin-Wave-Based Reservoir Computing

Wave-based systems are promising candidates for physical reservoirs in ML tasks such as robotics. However, water waves are naturally limited in practicality due to their macroscopic scale and environmental sensitivity. Moving beyond the proof-of-principle demonstrator, we explored (via simulations) the potential of SW RC, which offer nanoscopic, fast, reliable, and energy-efficient computation, along with nonlinear dynamics resembling those of water waves. To emulate the water-tank setup, we used SWs confined within a circular reservoir (diameter 1 μm), composed of a thin ferromagnetic film (thickness 1.5 nm).

On this micromagnetic scale, SWs manifest as oscillations in the magnetization. Analogous to water surface-wave dispersion, which depends on the balance of surface tension and gravity, SW dispersion is parameterized by the spin-exchange stiffness A_{ex} and external magnetic field B_{ext}. However, unlike water waves, the dynamics of the SW reservoir can be tuned by altering B_{ext} relative to its saturation magnetization (M_{sat}) and A_{ex}, thereby tuning the nonlinear properties of the reservoir. In our simulations, we modelled a typical CoFeB heterostructure with M_{sat} of 1 MA/m and A_{ex} = 15 pJ/m.

For low amplitudes, SWs exhibit a nearly linear dispersion; however, as the amplitude increases, nonlinear effects arise due to the larger precession angle of the spins, causing deviations from harmonic motion. This nonlinearity is central to their utility in RC, where complex dynamics and memory effects, often referred to as the echo-state property[2,3], are required for information processing. By tuning B_{ext} along with the frequency and amplitude of waves used to encode the input information, the reservoir parameters can be optimized for a diverse range of problems. SW-based computational systems are therefore strong candidates for universal learning machines, well-suited for robotics applications such as audio/visual recognition, autonomous motion, and fine motor control.

S5. Simulation Procedure for Spin-Waves

The GPU-accelerated software MuMax3[4] was employed to simulate SW magnetization dynamics via finite element approximation of the Landau-Lifshitz-Gilbert (LLG) equation. Although SWs can be excited using a magnetic field, electrical generation is much more practical for device applications. We therefore used the voltage-controlled magnetic anisotropy (VCMA) effect for input excitation: voltage pulses locally alter the magnetic anisotropy of the disk (600–800 fJ/Vm)[5] at specific transduction regions, launching SWs that propagate radially. Analogous to the water-tank setup, sensory data from the robot were encoded as wave inputs (f_{in} ~ 1 GHz) with time steps Δt = 200 fs, applied to six circular transduction regions (diameter 50 nm), as shown in Fig. 5a of the main text.

The simulations[6] captured SW propagation and interference within the reservoir over 4 ns. During this interval, the out-of-plane magnetization (m_z) profile of the disk was recorded in 200 frames using a strobe period of 20 ps. An example of a simulated image of the entire reservoir with input electrodes is shown in Fig. 5a of the main text. For faster analysis, data from the central square region of the disk (width 700 nm) were converted into 50 × 50 arrays, capturing the key wave features whilst reducing the memory requirement. They were then normalized to zero mean and unit variance to improve SVM performance[7].

Similar to the water-waves, the ML component of this RC setup involved training a SVM on the normalized wave amplitude outputs. The study into the spatial information requirements was

repeated for this setup; however, given that the simulation work was computationally intensive, the temporal study was not repeated (only the first few ns of evolution were required to solve the task, but the SW decay time was much longer than this period).

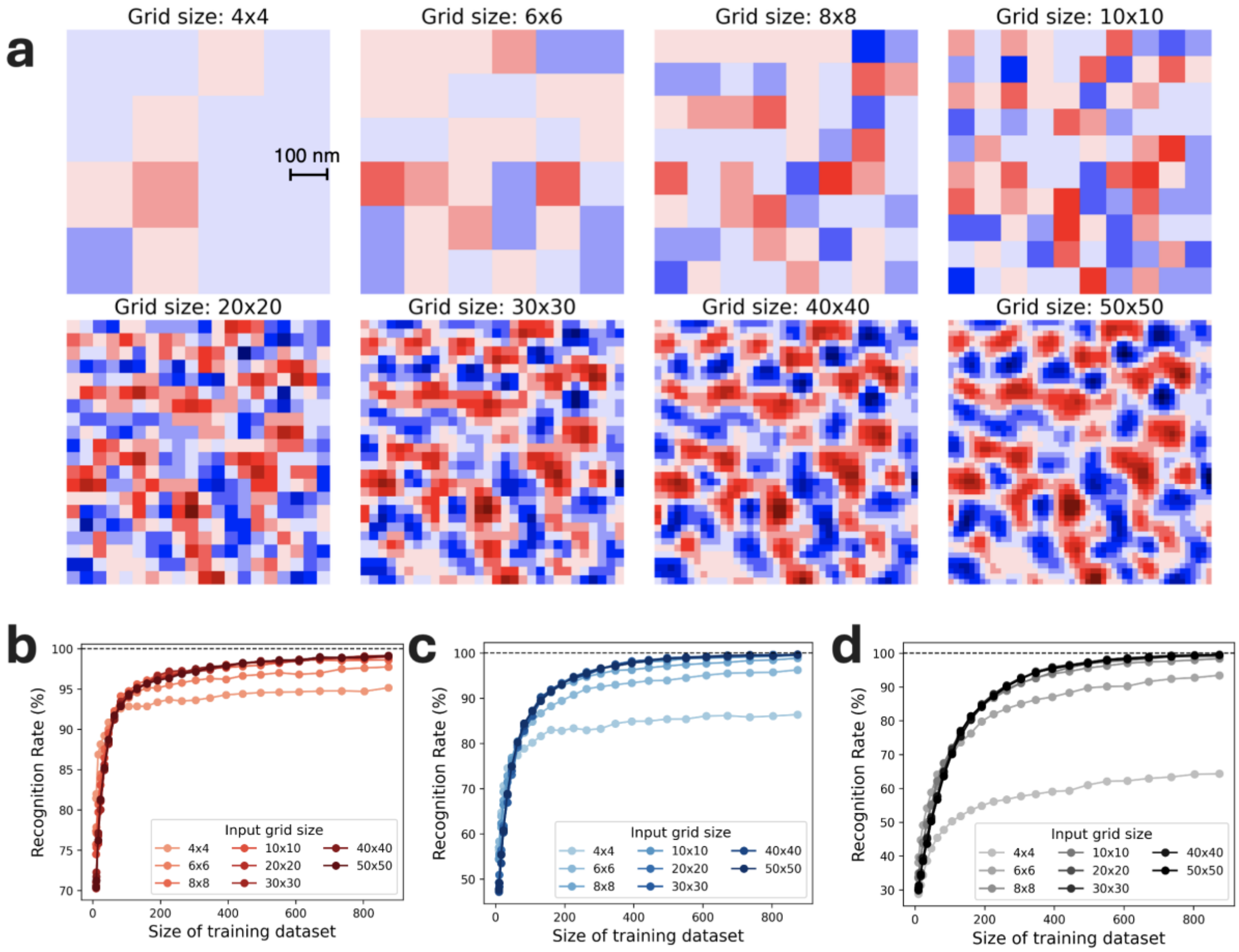

Supplementary Fig. 4. Optimization of output training for SW-based RC. (a) *Increasing the spatial resolution of the reservoir captures finer details, and therefore more information about the waveform. However, above grid sizes of ~30 × 30, the additional cells only add redundant information as the structural details of the wave are already reasonably well captured. The recognition accuracy increases with dataset size and it saturates at task-dependent values. For the SW reservoir, results for the* ***(b)*** *2-output,* ***(c)*** *3-output, and* ***(d)*** *5-output classification tasks are shown. In each case, performance is averaged over multiple training runs, and demonstrates that smaller grids (4 × 4, 6 × 6) reduce accuracy by obscuring spatial detail, while at higher resolutions (>8 × 8) performance saturates.*

The spatial grid study (similar to the water-wave study explained in Supplementary Fig. 3b) yielded almost identical results to those of the water reservoir, with the recognition rate saturating after the features of the wave become sufficiently resolved above a grid size of around 8 × 8 (Supplementary Fig. 4b-d). It is interesting to note that, compared with the water-wave case, a very fine image gridding was not required to achieve maximum accuracy. The reason is that the features of spin-wave interference are much richer than those of water-wave interference (Supplementary Fig. 5), allowing maximum accuracy even with modest gridding.

In all three classification tasks, the accuracy reached 100%, as shown in Fig. 5b of the main text. Although this result demonstrates the feasibility of achieving collision-free robotic motion, we did not perform real-time robotic operation with the simulated spin-wave RC, unlike in the water-wave case. This is because the simulation of a single input takes several minutes, making real-time control impractically slow. However, as discussed in the main text, a real spin-wave RC device would perform computation on the nanosecond scale, enabling real-time operation with ultrafast decision-making.

S6. Comparison between Spin-Wave and Water Wave RC

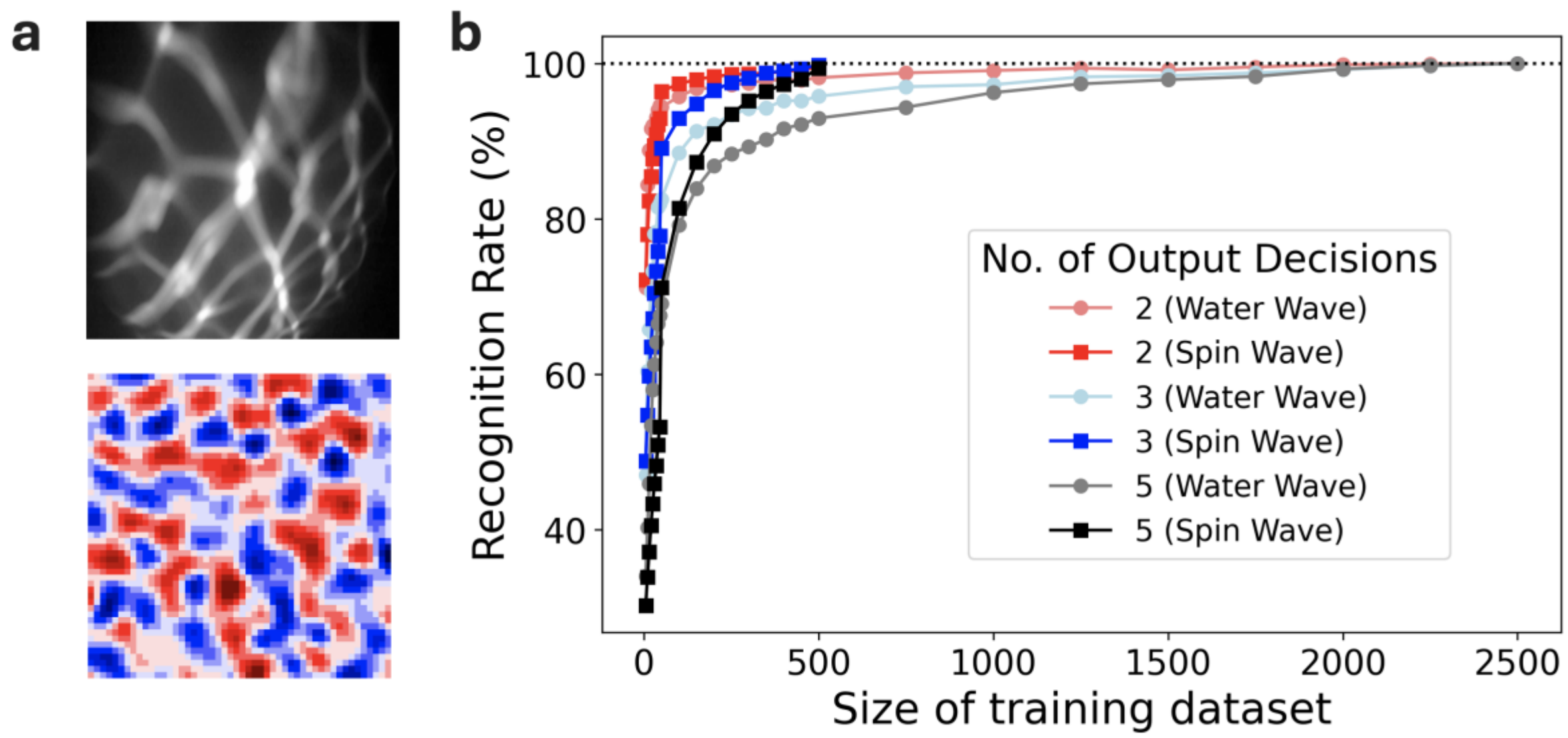

***Supplementary Fig. 5: Comparison of SW and water wave RC performance**: **(a)** Example of output interference images for water waves (top) and SWs (bottom) typically used for training. **(b)** Recognition rate as a function of the number of training samples for the 2-, 3-, and 5-output decision-making tasks using both SWs and water waves. Less training was required to achieve maximum accuracy with SWs compared to water waves for all tasks, likely due to the richer spatial features of the SW interference images, as shown in (a).*

S7. Results Using the MTJ Readout Method for Spin-Waves

For a real SWRC device, magnetic tunnel junctions (MTJs) could be fabricated on top of the reservoir to read out the magnetization profile information over time[8,9]. To emulate this within our simulation, 64 output regions were chosen, located in 8 concentric rings within the reservoir (Supplementary Fig. 6a), and only the data from these regions were used to train the SVM. Given that these regions were distributed on the same characteristic scale as the waveform, the SVMs trained on output features from the regions perform well (Supplementary Fig. 6b), with the information contained and thus performance comparable to that of an 8 × 8 grid.

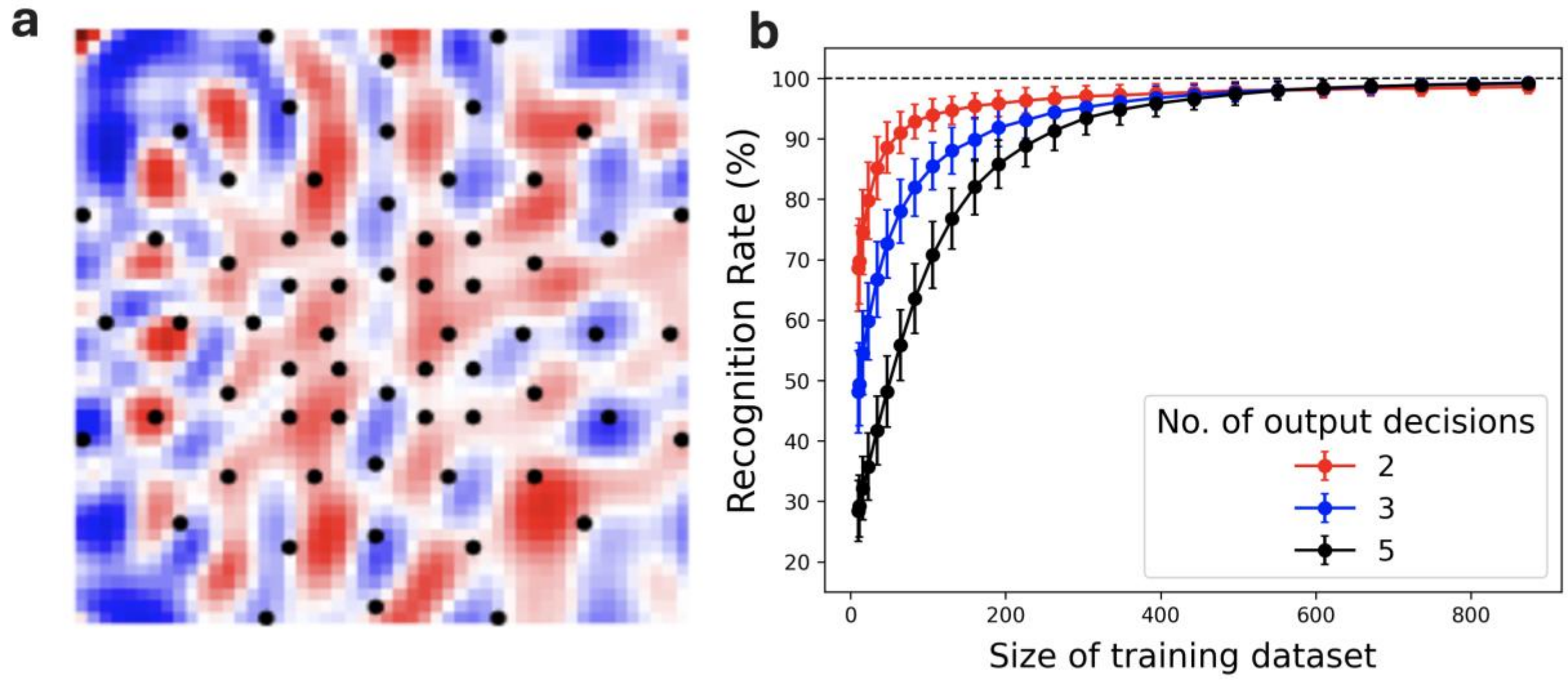

Supplementary Fig. 6. SW RC using an MTJ-based readout mechanism. (a) *The location of 64 output regions representing MTJ stacks is shown within the SW reservoir. They are spaced out at the vertices of 8 concentric octagons.* ***(b)*** *The recognition rate obtained by training the output features from these regions is plotted against the number of training samples for the 2-, 3-, and 5-output classification tasks. The results are comparable to those obtained by training on the full output grids, demonstrating the technological feasibility of the MTJ read-out approach for SW-based RC.*

S8. Quantitative Comparison of Spin-Wave RC with State-of-the-Art Neuromorphic Hardware

To substantiate our claim that wave-based RC offers a scalable and energy-efficient alternative to conventional neuromorphic architectures, we provide here a quantitative comparison of the proposed spin-wave RC platform against five representative state-of-the-art neuromorphic systems: two CMOS-based spiking neural network (SNN) chips (Intel Loihi[10] and IBM TrueNorth[11]), a memristor crossbar network[12, 13], a spintronic nano-oscillator RC system[14], and a photonic RC system[15]. The comparison is summarised in Supplementary Table 1.

The key architectural advantage of wave-based RC lies in its separation between the computational reservoir and the trainable output layer. Unlike weight-programmed architectures, in which all synaptic connections must be iteratively updated during training, the wave reservoir is a fixed physical system whose nonlinear dynamics emerge passively from wave interference. Only the linear output layer is trained using SVM, and as demonstrated in Supplementary Fig. 6, in our simulations, MTJ readout nodes were sufficient to achieve 100% classification accuracy across all tasks. This reduces the need for weight-programming circuitry (analogue-to-digital converters, write drivers, selectors), which constitute a major hardware overhead in passive memristive crossbar implementations[13], and avoids the large on-chip SRAM and dedicated learning units that contribute significantly to the area and power budgets of CMOS spiking chips[10,11].

In terms of operating speed, the spin-wave RC is capable of operating at GHz frequencies, placing it on a par with photonic RC systems and several orders of magnitude faster than clocked SNN implementations such as TrueNorth, while Loihi 2 uses a different event-driven architecture and

should be compared separately. The reservoir device footprint, a 1 μm diameter ferromagnetic disk with 50 nm transduction regions, is among the most compact of all platforms considered. Crucially, the CoFeB/MgO heterostructure is fully compatible with standard CMOS back-end-of-line processing, with MTJ stacks of comparable geometry already demonstrated at industrial scale in 28 nm fully depleted silicon-on-insulator (FDSOI) technology[21].

Regarding energy consumption, the reservoir itself is passive between input events and therefore consumes no dynamic power during idle periods, a property that underpins the event-driven operation mode demonstrated in Fig. 4d of the main text, where computation is triggered only upon changes in sensory input. An estimate of the input-excitation energy can be derived from the VCMA efficiency[5]; however, a comprehensive device-level energy benchmark including readout and interface circuitry requires a fabricated prototype and is beyond the scope of this simulation study. We note that a similar limitation applies to other physical RC platforms in the literature, where reported energy figures often exclude peripheral circuitry[15]. We have therefore included appropriate limitations in the footnotes of Supplementary Table 1.

Compared specifically with other RC platforms, wave-based RC possesses several distinguishing characteristics. Unlike time-multiplexed reservoirs, wave reservoirs naturally process information through both spatial and temporal degrees of freedom, generating a high-dimensional feature space through wave propagation and interference within a single physical layer. In addition, wave propagation, reflections, and finite decay times provide an intrinsic source of fading memory without requiring engineered delay lines or feedback loops. For spin-wave implementations, the reservoir dynamics can also be reconfigured after fabrication through external magnetic fields and excitation conditions while maintaining compatibility with CMOS-integrated spintronic devices. Together, these features provide a compact, programmable, and potentially energy-efficient platform for physical reservoir computing.

In summary, the spin-wave RC combines GHz-speed operation, nanoscale footprint, minimal hardware overhead, and a simple single-layer training protocol into a platform that is distinctly advantageous relative to weight-programmed architectures for embedded robotic applications.

Metric	CMOS-Loihi	CMOS-TrueNorth	Memristor Crossbar	Spintronic Nano-oscillator RC	Photonic RC	Spin-Wave RC (This Work)
**Operating frequency / computation speed**	Up to 5000x faster than biological neurons[10]	1 kHz tick rate; 46 GSOPS/W [11]	~μs–ms switching speed per device[12]; array inference speed limited by peripheral circuitry[12,13]	~100 MHz–1 GHz (STNOs)[14]	~kHz (SLM-based[15]; up to GHz–THz and 20 TOPS in integrated photonic RC[16] [e]	**~1 GHz (VCMA excitation); ~4 ns computation window**
**Energy per operation**	~20-50 pJ/synaptic op for Loihi 1[17]	~26 pJ/synaptic op[11,18]; 65 mW total chip power[11]	~pJ/op per device switching event [12] [d] ; ~400% peripheral programming overhead[13]	~24 fJ/neuron (vortex STNO)[19]	~4.4 fJ/op (state-of-the-art photonic RC) [17]	**~fJ range (VCMA: 600-800 fJ/Vm [b]); the reservoir itself consumes no dynamic power while idle; total**

Metric	CMOS-Loihi	CMOS-TrueNorth	Memristor Crossbar	Spintronic Nano-oscillator RC	Photonic RC	Spin-Wave RC (This Work)
						**energy is dominated by excitation and readout circuitry.**
**Hardware overhead (peripheral circuitry)** [c]	High: 128 neuromorphic cores + 192 KB SRAM/core; programmable microcode learning engine; dedicated on-chip learning circuits[10]	High: 5.4B transistors; weight-loading infrastructure required[11]	Very high: ~400% peripheral overhead for DAC/ADC, selectors and write drivers[13]	Medium: external microwave source and frequency-detection circuitry[14]	Medium: laser source, optical modulators, detectors, ADC [15]	**Low: single planar ferromagnetic film; MTJ readout nodes sufficient; no weight-programming circuitry**
**Device footprint**	Process: Intel 4; Die Area: 31 mm²; Core Area: 0.21 mm²; Transistors: 2.3 billion[10]	~4.3 cm^2 die area (Samsung 28 nm) [11]	~um2 per crosspoint; 3D integration required for density[12]	~100 nm-um per oscillator[14]	cm-scale (free-space/fibre, off-the-shelf setup) [15]; mm-scale (integrated photonic chip)[16] [f]	**1 μm diameter reservoir; 50 nm transduction regions**
**Trainable parameters**	~1 million neurons (128 cores x 8,192); ~120 million synapses;[10]	256 M synapses (pre-trained, fixed at runtime) [11]	Entire conductance weight matrix (thousands-millions of states)[12,19]	Output layer only (RC paradigm)[14]	Output layer only (RC paradigm) [15]	**Output layer only (RC paradigm)**
**Training approach**	On-chip programmable learning (STDP, three-factor rules); Lava framework for off-chip coordination[10]	Off-chip pre-training; weights loaded statically [11]	Backpropagation/gradient-based; weight programming per update[12]	Linear regression/SVM on output layer only[14]	Linear regression/SVM on output layer only [15]	**SVM on output layer only; no backpropagation; no reservoir weight tuning**
**Fabrication complexity**	Very high (custom Intel 4 EUV process;	Very high (custom IBM process;	High - 3D crossbar integration; device	Medium - nanofabricated pillars;	Moderate (off-the-shelf components) [15]; High for	**Medium - single planar ferromagnetic films with**

Metric	CMOS-Loihi	CMOS-TrueNorth	Memristor Crossbar	Spintronic Nano-oscillator RC	Photonic RC	Spin-Wave RC (This Work)
	2.3B transistors; 31 mm² die)[10]	5.4B transistors)[11]	uniformity challenging[12]	microwave circuits[14]	integrated chip - photonic chip or fibre cavity; laser and detector integration [16]	**MTJs; standard thin-film deposition and lithography**
**Weight reconfiguration required**	Yes - synaptic weights updated during/after training[10]	Yes - weights pre-programmed before deployment [11]	Yes - resistive state of each crosspoint must be programmed[12]	No - reservoir fixed; only output layer trained[14]	No - reservoir fixed; output layer trained externally [15]	**No - reservoir requires no reconfiguration; tunable via external field B_{ext} if desired**
**Primary task demonstrated**	Speech/image recognition; Keyword spotting, sparse coding, gesture recognition, optical flow estimation[10]	Image classification [11]	Simple pattern classification (3×3 pixel images of letters)[12]	Waveform/speech classification[14]	Human action recognition; signal classification [15]	**Robotic obstacle recognition (2, 3, 5-output classification); 100% accuracy)**

Supplementary Table 1. *Quantitative comparison of the proposed spin-wave RC platform with representative state-of-the-art neuromorphic hardware. The spin-wave RC column (highlighted in blue) corresponds to the simulated CoFeB device described in this work. All figures for comparison platforms are drawn from the cited primary literature. Energy figures across platforms reflect different task complexities and measurement conventions; direct cross-platform comparisons are therefore indicative rather than definitive (see footnotes below).*

a. Energy figures reflect different task complexities and levels of peripheral circuitry inclusion across platforms. Cross-platform comparisons are indicative.

b. The energy estimate for spin-wave RC is based on the VCMA excitation efficiency (600–800 fJ/Vm, ref. [5]) and does not include readout or peripheral circuitry; full device-level benchmarking requires a fabricated prototype.

c. 'Hardware overhead' for RC platforms (spintronic, photonic, spin-wave) refers to output-layer and readout circuitry only, as the reservoir itself requires no weight-programming infrastructure.

d. The ~pJ/op energy figure for the memristor crossbar is an estimate derived from the set/reset pulse parameters reported in the Supplementary Information of Prezioso et al. (ref. [12]): set pulse = 2 V, 500 µs; reset pulse +2 V, 500 µs. No explicit energy-per-operation figure is stated in the main text of ref. [12

e. The approximately kHz operating rate reported for the photonic RC in ref. [15] reflects the frame rate of the spatial light modulator used in that off-the-shelf free-space setup (pp. 531-532). By contrast, the GHz to THz bandwidth, 20 TOPS throughput, and 4.4 fJ/op energy efficiency reported in ref. [16] refer to an integrated all-optical photonic RC based on a nonlinear amplifying loop mirror (NALM) fibre cavity. These figures of merit therefore correspond to qualitatively different system implementations and should be interpreted accordingly.

f. The cm-scale footprint refers to the free-space optical bench in ref. [15] . The mm-scale and high-fabrication-complexity assessments apply to integrated photonic RC chips (ref. [18]). The CMOS compatibility limitation (III-V or SiPh platforms;

electro-optic conversion required) applies to chip-integrated photonic RC and is not discussed in ref. [14], which uses off-the-shelf components.

These benchmarking comparisons were performed based on the currently available information for the different technologies, which may evolve over time. Therefore, the comparisons presented in this section should not be interpreted as universal rankings between neuromorphic approaches, but rather as indicative comparisons for the specific task classes and implementation assumptions considered in this work.

S9. Computational benchmarking

Within the field of RC, the reservoir is typically treated as a black box with an internal dynamical state that is inaccessible, the output is merely a small subspace of the hidden information state (small by comparison to the total state, but still sufficiently high-dimensional for task completion). This makes optimising such systems challenging, and unlike traditional NNs there is no possibility for gradient-based regression. Despite this, there are a few metrics that are widely used and generally capture the main qualities which make a good reservoir: the kernel rank (KR) directly measures the effective number of linearly independent features the reservoir output contains and is thus a metric for its dimensionality. Finally, by training a diverse set of NNs and the SWRC on a typical nonlinear classification problem (such as XOR), we gain insight into the level of complexity (and thus the equivalent volume of compute) with which our system's internal dynamics operate.

S9.1 Kernel Rank

To calculate the kernel rank (KR) of the feature space, we construct a feature matrix

$$\Psi = [\, \psi_1{}^{\mathrm{T}} \,;\, \psi_2{}^{\mathrm{T}} \,;\, \vdots ;\, \psi_{\mathrm{n}}{}^{\mathrm{T}} \,] \in \mathbb{R}^{\mathrm{NxT}}$$

where ψ_t denotes the flattened reservoir output (feature vector) at time t, X, T are the number of spatial/temporal features per output, and N is the number of samples. The KR is the rank of this matrix and provides a measure of the reservoir's effective dimensionality. To probe this further, we perform a singular value decomposition (SVD)

$$\Psi = U\,\Sigma\,V^{\mathrm{T}} \ .$$

Then we calculate the cumulative explained variance, given by

$$C(r) = (\Sigma_{\mathrm{i=1}}{}^{\mathrm{r}}\, \sigma_{\mathrm{i}}{}^{2}) / (\Sigma_{\mathrm{i=1}}{}^{\mathrm{min}}(X \times T, N)\, \sigma_{\mathrm{i}}{}^{2}) \ .$$

We define the d95 as the smallest integer r such that

$$d95 = C(r) \geq 0.95 \, .$$

Since the maximum achievable rank is bounded by min ($X \times T$, N). In this work, the feature dimension $D = X \cdot T$ greatly exceeds the sample count N at every coarse-graining scale, so this bound is fixed at $N - 1 = 255$. To resolve how the variance is distributed across the retained dimensions we additionally report the participation ratio,

$$PR = \frac{(\Sigma_i \sigma_i^2)^2}{\Sigma_i \sigma_i^4} \ ,$$

a continuous, variance-weighted effective rank: PR is large when the variance is spread evenly across many modes and small when it is concentrated in a few.

We computed KR, d95 and PR for each coarse-grained feature set in Supplementary Fig. 4, from spatial grids of 50 × 50 down to 4 × 4 (see Table S9.1 and Fig. S9.2). The feature matrix Ψ_c is full rank, at the numerical limit $N - 1 = 255$ at every coarse-graining scale, confirming a high-quality separable feature map. The d95 value is also broadly constant with spatial resolution: it remains at 25 to 26 for the four finest grids down to 10 × 10, where it then decreases slightly. This means that 95% of the variance is carried by only about 20 to 26 modes, largely independent of spatial resolution, which indicates that the key discriminating information is concentrated in a small number of large-scale spatial modes that survive coarse-graining.

**Grid**	$D = X \cdot T$	**d95**	**PR**	**Rank (Ψc)**
50 × 50	502, 500	26	24.3	255
40 × 40	321, 600	26	24.1	255
30 × 30	180, 900	26	24.1	255
20 × 20	80, 400	26	23.7	255
10 × 10	20, 100	25	20.6	255
8×8	12, 864	23	17.2	255
6 × 6	7,236	19	13.4	255
4 × 4	3,216	22	8.4	255

Supplementary Table 2. *Kernel rank, d95 and participation ratio of the reservoir feature matrix across spatial coarse-graining scales; the numerical rank of the feature matrix is 255 = N − 1 at every scale, but the d95 and PR drop off once the coarse-graining moves beyond the Nyquist scale of the system, and thus the waveform is no longer fully resolved.*

The participation ratio resolves where coarse-graining begins to degrade the representation. PR is flat at ≈ 24 for grids down to 30 × 30, then begins to fall, mirroring the spatial resolution results shown in Supplementary Fig. 4. The reservoir therefore maintains a full-rank, input-separable feature space across a broad range of spatial resolutions, with a well-defined spatial-Nyquist limit revealed only by the variance-weighted rank.

S9.2 Computational Capacity

To assess the computational capacity of the SWRC from a complementary angle, we compare its performance on a standard nonlinear benchmark, XOR classification, with NNs of different architectures, including MLPs and CNNs of various widths and depths. This allows us to estimate the compute volume, that is, how many floating-point operations (FLOPs) the SWRC requires relative to the corresponding MLP or CNN. Because the SWRC system only uses a linear SVM readout in the output layer, its compute cost is concentrated in the readout, whereas conventional NNs require backpropagation through all layers over many epochs.

For a linear SVM operating on a feature vector of dimension K, the inference cost is given by

$$F_{SVM,pred} = 2K$$

This is the standard leading-order FLOP count for a dense linear model[1,21]. Following LIBLINEAR-style dual coordinate-descent optimisation, the training cost scales approximately linearly with the number of training samples and features:

$$F_{SVM,train} \approx c\, n_{iter}\, N_{train}\, K, \qquad c \approx 3$$

where n_{iter} is the number of optimisation sweeps through the training set and c is an empirical prefactor reflecting the average coordinate-update cost.

For an MLP of depth d, hidden width w, input dimension D, and output dimension C, the forward-pass FLOP count is approximated by[22]

$$F_{fwd} = 2(Dw + (d-1)w^2 + wC)$$

Assuming standard backpropagation, the total training cost over E epochs become

$$F_{MLP} = 3\, F_{fwd}\, N_{train}\, E \ ,$$

using the common approximation that backward propagation requires approximately twice the compute of a forward pass.

For CNN architectures using $k \times k$ convolutions with width w and depth d, the forward FLOP count per output pixel is

$$F_{fwd,CNN}^{pix} = 2(D_{in} w k^2 + (d-1) w^2 k^2 + wC)$$

and therefore, the total training cost over a full $H \times W$ image is

$$F_{CNN} = 3\, F_{fwd,CNN}^{pix}\, H \cdot W \cdot E$$

The expressions above therefore provide leading-order estimates for the relative compute volume of the SWRC system compared to conventional neural-network baselines.

The XOR (exclusive OR) task is used as a standard benchmark within ML, providing a textbook example of a linearly inseparable problem. The solution is the parity of the bit-string, and no trivial decision boundary (a straight line in this case) can separate the even and odd classes in the input space. However, a NN with just one hidden layer and nonlinear activations can solve XOR for a 2 bit-string. The hidden layer maps the input into a space where the problem becomes linearly separable, which is why XOR is a standard benchmark for testing nonlinearity.

We extend the XOR decision boundary to finer chequerboard grids to create problems of increasing difficulty, which are displayed in Supplementary Fig. 7. Given the oscillatory nature of spin waves, the modes of the SWRC form a basis set that decomposes functions through the lens of periodicity; function approximation in this system is reminiscent of Fourier decomposition. Naturally, it performs well on problems where the solution has a geometry of repetition. With very few features, it is able to well approximate the XOR decision boundary. Given the oscillatory nature of spin waves, the reservoir modes provide a basis of periodic features, so function approximation in this system is reminiscent of Fourier-like decomposition. Naturally, it performs well on problems where the solution has a geometry of repetition. With very few features, it can already approximate the XOR decision boundary.

The caveat is that this system performs well when the problem is set up in a way that makes harmonic-like analysis sensible, with data features that contain periodic elements, such as the XOR chequerboard pattern. However, given the prevalence of harmonic analysis within data science/ML

(especially in spatial awareness problems that are common in robotics), it is fair to say that a reservoir that uses an inherently oscillatory basis could have widespread application potential. One additional insight here is that the performance of the SWRC on this XOR task matches with the resonance scale of encoded pulses ($f \sim 1$ GHz). In Supplementary Fig. 7 we see the SWRC performance peaks between $n = 2$ and 3, as the variation in the problem geometry matches the oscillation scale of the system. This suggests that performance depends on matching the task's spatial scale to the reservoir's characteristic oscillation scale, so other task geometries may benefit from retuning the driving frequency.

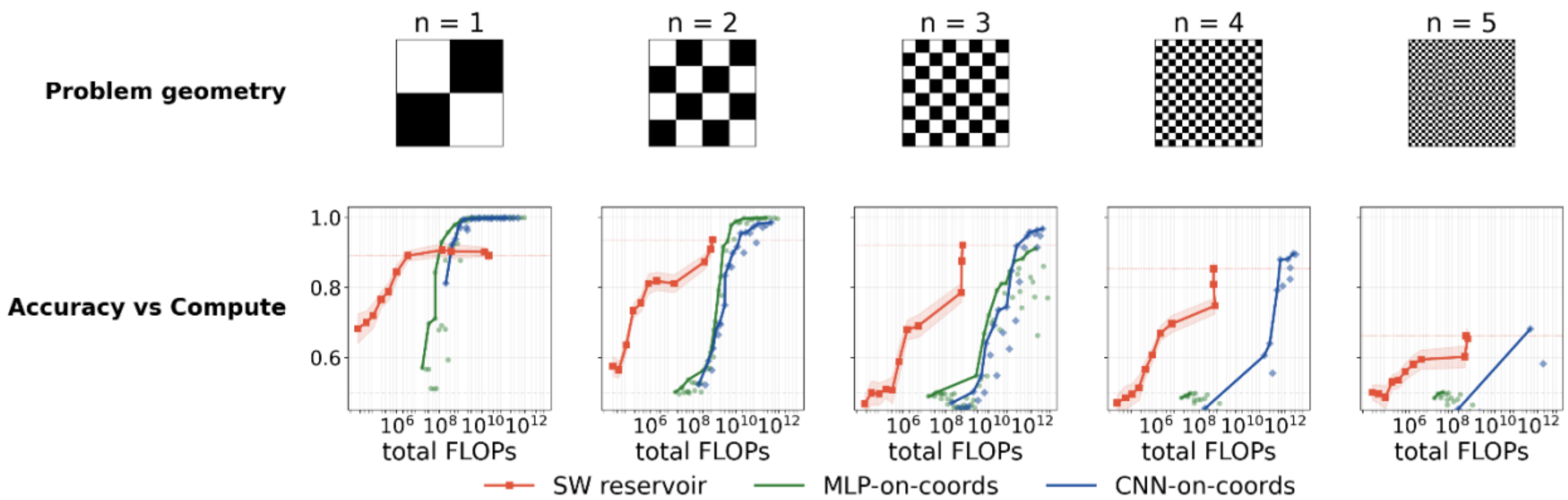

**SW target**	0.892	0.937	0.921	0.854	0.662
**Smallest model**	MLP d1w16	MLP d4w32	CNN d8w64	CNN d8w64	CNN d6w64
**Parameters**	82	3,330	259,842	259,842	185,986
**Model accuracy**	0.931	0.978	0.948	0.880	0.681

Supplementary Fig. 7. Comparison of the SWRC to MLPs and CNNs on an XOR task. *This figure is split into 5 columns, each showing the performances of the different ML models on XOR decision boundary tasks of increasing difficulty. The tasks are defined on a 32×32 grid, with the class output being the N-bitwise XOR of the 2D coordinate's bitstring. This creates a chequerboard-like decision boundaries which are displayed in the problem geometry row. The efficiency of physical RC is clear from the plots in the Accuracy vs. compute row, with the SWRC achieving accuracies comparable to MLPs/CNNs that use several orders of magnitude more compute (measured in total training and inference FLOPs) as they are trained over 400 epochs. Large enough NNs outperform the SWRC (for the P=2,1 task, the MLP is computationally unable to resolve the decision boundary at all but, the CNN works in all cases), but their complexity reaches hundreds of thousands of parameters as shown in the table in the bottom row.*

S10. Supplementary Videos Description

Supplementary Video 1

Autonomous motion of a virtual robotic car performing obstacle avoidance controlled by wave reservoir computing. A Pygame-based car navigates in a virtual room containing obstacles of various shapes. Every 500 ms, a sensor (blue dotted line) measures distances at six angles, which are sent to the water-wave reservoir to generate input waves. The resulting interference pattern is evaluated by the trained model to command forward motion or turning. Frames were compiled into a 30-frames-per-second video. The robot's motion path is summarized in Fig. 4b.

Supplementary Video 2

Autonomous motion of an Arduino-based robotic car performing obstacle avoidance controlled by wave reservoir computing. The Arduino-based car navigates a 3 m × 2 m obstacle course enclosed by wooden boundaries. The car is equipped with an ultrasonic sensor that measures distances and sends them via Bluetooth to excite the reservoir input; manoeuvre commands are then transmitted back through the same channel. To create a compact presentation, the real-time video was sped up 50×. The robot's motion path is summarized in Fig. 4c.

Supplementary Video 3

Event-based operation of the virtual car. A transistor, whose state is indicated by red or green LEDs, is integrated into the virtual car. The car moves continuously without computation when the transistor is off and activates computation only when changes occur in the incoming distance-sensor data. At those moments, the car moves according to the wave-computing decisions. The absence of continuous computation allows fast motion in open space, while more cautious maneuvers occur near obstacles, demonstrating the environmental adaptability of event-based robotic control. The robot's motion path is summarized in Fig. 4d.

References:

1. Cervantes, J., Garcia-Lamont, F., Rodríguez-Mazahua, L. & Lopez, A. A comprehensive survey on support vector machine classification: Applications, challenges and trends. *Neurocomputing* **408**, 189–215 (2020).
2. Tanaka, G. *et al.* Recent advances in physical reservoir computing: A review. *Neural Netw.* **115**, 100–123 (2019).
3. Marcucci, G., Pierangeli, D. & Conti, C. Theory of neuromorphic computing by waves: Machine learning by rogue waves, dispersive shocks, and solitons. *Phys. Rev. Lett.* **125**, 093901 (2020).
4. Vansteenkiste, A. *et al.* The design and verification of MuMax3. *AIP Adv.* **4**, 107133 (2014).
5. Safeer, C. K. *et al.* Magnetization dynamics driven by displacement currents across a magnetic tunnel junction. *Phys. Rev. Appl.* **22**, 024019 (2024).
6. J. Zohar, *jacobzohar/wave-based-neuromorphic-hardware: First release* (Version v1.0.0). Zenodo. https://doi.org/10.5281/zenodo.21772974 (2026).
7. Hsu, C.-W., Chang, C.-C. & Lin, C.-J. *A practical guide to support vector classification.* Available at https://www.csie.ntu.edu.tw/~cjlin (2003).
8. Heins, C. *et al.* Electrical detection of magnons with nanoscale magnetic tunnel junctions. *arXiv* 2509.19483 (2025).
9. Pinna, D., Bourianoff, G. & Everschor-Sitte, K. Reservoir computing with random skyrmion textures. *Phys. Rev. Appl.* **14**, 054025 (2020).
10. Davies, M. *et al.* Advancing neuromorphic computing with Loihi: a survey of results and outlook. *Proc. IEEE* **109**, 911–934 (2021); Intel Labs. Loihi 2: taking neuromorphic computing to the next level. Intel Technology Brief https://www.intel.com/content/www/us/en/research/neuromorphic-computing-loihi-2-technology-brief.html (2021).
11. Akopyan, F. *et al.* TrueNorth: design and tool flow of a 65 mW 1 million neuron programmable neurosynaptic chip. *IEEE Trans. Comput.-Aided Des. Integr. Circuits Syst.* **34**, 1537–1557 (2015).
12. Prezioso, M. *et al.* Training and operation of an integrated neuromorphic network based on metal-oxide memristors. *Nature* **521**, 61–64 (2015).
13. Choi, S. *et al.* Wafer-scale fabrication of memristive passive crossbar circuits for brain-scale neuromorphic computing. *Nat. Commun.* **16**, 9013 (2025).
14. Torrejon, J. *et al.* Neuromorphic computing with nanoscale spintronic oscillators. *Nature* **547**, 428–431 (2017).
15. Antonik, P., Marsal, N., Brunner, D. & Rontani, D. Human action recognition with a large-scale brain-inspired photonic computer. *Nat. Mach. Intell.* **1**, 530–537 (2019).
16. Aadhi, A. *et al.* Scalable photonic reservoir computing for parallel machine learning tasks. *Nat. Commun.* **16**, 67983 (2025).

17. Davies, M. et al. Loihi: A Neuromorphic Manycore Processor with On-Chip Learning. IEEE Micro 38, 82–99 (2018)

18. Merolla, P. A. *et al.* A million spiking-neuron integrated circuit with a scalable communication network and interface. *Science* **345**, 668–673 (2014).

19. Shreya, S. *et al.* Granular vortex spin-torque nano oscillator for reservoir computing. *Sci. Rep.* **13**, 16632 (2023).

20. Grollier, J. *et al.* Neuromorphic spintronics. *Nat. Electron.* **3**, 360–370 (2020).

21. K. Lee *et al.*, 1 Gbit high density embedded STT-MRAM in 28 nm FDSOI technology. *IEEE Int. Electron Devices Meet. (IEDM.*, 19–23 (2019).

22. *scikit-learn developers.* LinearSVC documentation. Available at https://scikit-learn.org/stable/modules/generated/sklearn.svm.LinearSVC.html

23. Goodfellow, I., Bengio, Y. & Courville, A. *Deep Learning* (MIT Press, 2016).
