# Supplementary material for: Autonomous robotic operation controlled by wave-based neuromorphic hardware
Source: Nat Commun. 2026 Sep 10;17:10677. doi: 10.1038/s41467-026-77661-3 (PMC13654935; doi:10.1038/s41467-026-77661-3)
Supplement: Supplementary file 2 [file 41467_2026_77661_MOESM2_ESM.pdf]

Description of Additional Supplementary Files

Supplementary Video 1. Autonomous motion of a virtual robotic car performing obstacle avoidance controlled by wave reservoir computing.

Supplementary Video 2. Autonomous motion of an Arduino-based robotic car performing obstacle avoidance controlled by wave reservoir computing.

Supplementary Video 3. Event-based operation of the virtual car.
