# Supplementary material for: Autonomous robotic operation controlled by wave-based neuromorphic hardware
Source: Nat Commun. 2026 Sep 10;17:10677. doi: 10.1038/s41467-026-77661-3 (PMC13654935; doi:10.1038/s41467-026-77661-3)
Supplement: Supplementary file 3 [file 41467_2026_77661_MOESM6_ESM.pdf]

nature portfolio

Peer Review File

Autonomous robotic operation controlled by wave-based neuromorphic hardware

Corresponding Author: Dr C.K. Safeer

This file contains all reviewer reports in order by version, followed by all author rebuttals in order by version.

Version 0:

Reviewer comments:

Reviewer #1

(Remarks to the Author)
The authors present a wave-based reservoir computing (RC) system in which the reservoir is a body of water. An array of actuators launches propagating surface waves in a container, where the actuators' trigger times are encoded from discrete sensory readings (in this work, obstacle distance). Different obstacle states in the test field therefore produce different wave-interference patterns, which provide a nonlinear transformation into a higher-dimensional representation space. The resulting wave-field images are then classified and linearly mapped to command classes for controlling a robotic rover. The authors also investigate how temporal and spatial dynamics affect recognition accuracy, and they outline a prospective path toward chip-scale implementations by simulating an analogous scheme using nanoscale spin waves.
Overall, this is an interesting and potentially impactful contribution that could stimulate work in hardware-based AI, analog/edge computing, and neuromorphic computing and control. That said, several important questions should be addressed to clarify novelty, RC-specific functionality, and design choices:

A key advantage of reservoir computing is its ability to directly process spatiotemporal information in analog sensory streams. In the present work, however, the inputs are discrete distance readings that are first encoded into discrete actuator initiation times. Please clarify the intrinsic difference between this approach and previously reported spike-/event-encoded RC systems (e.g., Nature Electronics 4, 748–756 (2021)). In particular, what aspects of "wave-based" dynamics provide capabilities beyond a generic time-encoding front end followed by a dynamical medium?

Another core characteristic of RC is that the reservoir state depends not only on the current input but also on recent input history (fading memory), enabling context- and trend-dependent outputs. How does the proposed water-wave reservoir implement and quantify this property? For example, can the authors report memory capacity (or an equivalent metric), demonstrate performance on a task that explicitly requires temporal integration, or otherwise show that decisions depend on prior inputs rather than solely on instantaneous distance-to-spike timing?

What is the basis for the chosen spatial arrangement of the six actuators? Please discuss whether there is an optimal actuator spacing (or layout) for maximizing separability/accuracy, and how sensitive performance is to this parameter. If possible, include a sweep (or an argument) relating spacing to wavelength, container dimensions, boundary conditions, and interference richness.

The spin-wave simulations suggest characteristic operating frequencies in the GHz range. How would a spin-wave implementation interface with or be useful for controlling systems whose relevant dynamics are orders of magnitude slower (e.g., robotics, environmental sensing)? Please discuss possible strategies (e.g., temporal multiplexing, buffering/windowing, event-driven sampling, or multi-timescale reservoirs).

The proposed reservoir relies on coherent wave dynamics. Is coherence necessary for the claimed RC functionality, or is it mainly one possible physical substrate? Prior memristive RC demonstrations do not exhibit prominent coherent wave behavior yet still achieve RC effects. Additionally, recent work (e.g., Sci. Adv. 11, eadu2663 (2025)) shows high state richness in electrically tunable semiconductor grids without explicit wave coherence. Please clarify what unique neuromorphic/RC functions the present wave-based approach enables that are difficult to realize with those alternative reservoirs (e.g., specific advantages in controllable spatial modes, programmability, energy scaling, robustness, or task classes).

(Remarks on code availability)

Reviewer #2

(Remarks to the Author)
The paper by Zohar et al. presents a very interesting and potentially impactful case study of a physical reservoir computing system, implemented experimentally using water waves and explored via simulations using spin waves. While there is a substantial body of literature on physical reservoir computing and its computational capabilities, there are relatively few fully realized demonstrators—i.e., complete, built, and experimentally validated systems with all implementation details carefully addressed. In this regard, the present work is particularly valuable and timely. The authors demonstrate awareness of prior work on wave-based reservoir computing, and their hands-on, implementation-focused approach is highly appreciated. Overall, the study is well motivated and could provide an important contribution to the advancement of hardware-based reservoir computing.

However, despite these strengths, the manuscript currently lacks critical details and rigor required for publication in Nature Communications. There is no benchmarking against existing methods. The authors state that 100% accuracy can be achieved by selecting appropriate parameters, but this claim is presented without sufficient context or comparative evaluation. The chosen task—robotic navigation in a simple environment—is not particularly challenging, and the manuscript does not demonstrate the advantages of the proposed approach over conventional methods. The comparison is limited to a linear SVM, whereas more appropriate baselines, such as a simple multilayer perceptron (MLP) or convolutional neural network (CNN), would provide a fairer and more informative assessment. While the Supplementary videos are nice, they do not allow to quantify how difficult really the navigation task is.

Furthermore, it remains unclear whether the proposed approach offers any practical benefit in terms of training efficiency or computational cost. The manuscript provides very limited detail on how the reservoir output is trained, and there is no discussion of the associated computational complexity or resource requirements. This omission makes it difficult to evaluate the claimed advantages of the system, in the context of online training for robotic navigation.

The least developed and most technically vague part of the manuscript is the simulation study involving spin waves. Essential details are missing: the geometry of the system is not described, it is not even clear what material is used in the ferromagnetic thin film. The cited GitHub repository lacks documentation and is poorly commented. Moreover, for spin-wave systems to function as reservoirs, they must operate in a weakly nonlinear regime. There is not a single word in the paper how this is achieved, what are the applied power levels at the input.

Overall, the paper is valuable work and very much support the publication of hands-on results in this field. In its present shape, however, the paper does not meet the rigor and detail expected from. Nat. Comm. publication.

(Remarks on code availability)
Minimal comments, no documentation for the python script running micromagnetic code. Choice of material parameters is unclear and some appears to be arbitrary. The T=8ns simulation time seems practically challenging to implement. No examples are given, no example data files, no links to the paper material

The code is a useless resource at this point.

Reviewer #3

(Remarks to the Author)
This work proposes a wave-based reservoir computing (RC) platform for robotic control, demonstrating its potential through water-wave experiments and spin-wave simulations. The concept of utilizing physical wave interference as a scalable and energy-efficient alternative to digital neuromorphic hardware is scientifically interesting. However, the manuscript suffers from technical overstatements and a lack of rigorous justification. The term "navigation" is used loosely to describe what is essentially local obstacle avoidance, and the necessity of an RC framework for such low-complexity tasks remains unproven. Furthermore, the claim of an "event-driven" mechanism lacks supporting technical details, and the transition to "practical" spin-wave chips is premature given the acknowledged experimental hurdles. Without quantitative benchmarking against state-of-the-art hardware and the resolution of logical inconsistencies, I cannot recommend the paper for publication in its current form. The following issues must be addressed:
1. The authors frequently use the term "robotic navigation" throughout the text and in the title. However, the actual functionality demonstrated by the system aligns more accurately with local "obstacle avoidance" or purposeless "wandering." In the robotics community, "navigation" strictly implies the capability to answer "Where am I?" and "Where am I going?"—typically involving localization, mapping, and global path planning toward a specific goal. The authors are strongly advised to revise the terminology across the manuscript and the title (e.g., replacing it with "obstacle avoidance" or "reactive control") to accurately reflect the scope of the work and prevent misleading the readers.
2. There is an overstatement regarding the novelty of the experimental setup. The authors claim: "To overcome this, we first developed a unique experimental system based on water waves." However, as indicated by the authors' own reference [34] (Pattern recognition in a bucket), wave-based experimental setups for computation have been previously established. The authors should tone down absolute claims (e.g., removing "first developed") and objectively state the specific improvements

or unique aspects their system offers over existing water-wave platforms.
3. There is an inconsistency between Figure 2(f) and its corresponding caption. The text states "No object is the fifth class," but this description does not accurately correspond to the fifth condition visually presented in the figure. The authors should carefully check and revise the figure caption to ensure it perfectly matches the visual data, thereby avoiding reader confusion.
4. For the specific application scenario presented in the study, the necessity of an RC system is insufficiently justified. If the task predominantly involves simple wandering or basic obstacle avoidance, such low-complexity objectives can typically be achieved using direct sensor feedback or simple threshold-based reactive control. The current experiments fail to demonstrate the unique advantages of RC. The authors should either introduce more complex tasks (e.g., processing complex spatio-temporal dynamics) or provide a robust discussion detailing why the RC system provides an irreplaceable advantage over traditional sensor-direct-control paradigms in this specific context.
5. There is a logical contradiction regarding the applicability of spin waves. The authors claim that spin waves can meet the requirements for "practical on-chip robotic systems." However, later in the text, they acknowledge that the "interference and readout of multiple nanoscale spin waves remain active experimental challenges" and rely purely on micromagnetic simulations for quantification. If the experimental readout and interference are yet to be realized, it is premature to claim the system meets "practical" requirements. The authors should soften this claim, presenting the spin-wave approach as a promising future direction rather than an already viable, practical solution.
6. The manuscript mentions "sensory systems that trigger computation only upon dynamic changes in the environment." However, the technical details supporting this claim are missing. It is entirely unclear how this event-driven mechanism is physically or algorithmically realized using only a single transistor. The authors must provide a detailed explanation—and ideally a circuit logic diagram or operational principle schematic—to substantiate this claim.
7. The authors propose wave-based computation as a "scalable and energy-efficient alternative." To make this claim scientifically convincing, qualitative descriptions are insufficient. The manuscript requires a detailed, quantitative comparison (e.g., in a tabular format) between the proposed wavebased system and representative state-of-the-art neuromorphic devices. Metrics such as energy consumption, computational efficiency, and hardware overhead must be explicitly compared to substantiate the claimed advantages of the proposed approach.

(Remarks on code availability)

Version 1:

Reviewer comments:

Reviewer #1

(Remarks to the Author)
The authors have addressed my questions.

(Remarks on code availability)
The code for spin-wave-disk reservoir computing looks complete and reproducible for readers.

Reviewer #2

(Remarks to the Author)
The authors provided detailed answers to my concerns. I appreciate the added S8 and S9 , which give significantly more context to this work in the large literature of reservoir computing.

More details were added to the description of the machine learning methodology and important details now appear in the main article, not only in the supplementary material. The spin-wave part is, to my opinion is still somewhat hsnd-waving and built on analogies (the VCMA excitation mechanism, for example, not at all trivial), but I understand that it serves as a perspective for wave-based computing hardware and is not the central message of the paper.

Overall, I believe that the strengths of the work, particularly the value of demonstrating a fully implemented, hands-on reservoir computing system, significantly outweigh remaining flaws. I consider this to be a strong contribution, and the paper has the potential to become a highly cited article in Nature Communications.

(Remarks on code availability)
The GitHub repository is very nicely done, well documented!

Reviewer #3

(Remarks to the Author)
After reading this revised manuscript. all my questions have been addressed and i have no further question on the revised manuscript.

(Remarks on code availability)

Open Access This Peer Review File is licensed under a Creative Commons Attribution 4.0 International License, which permits use, sharing, adaptation, distribution and reproduction in any medium or format, as long as you give appropriate credit to the original author(s) and the source, provide a link to the Creative Commons license, and indicate if changes were made.
In cases where reviewers are anonymous, credit should be given to 'Anonymous Referee' and the source.
The images or other third party material in this Peer Review File are included in the article's Creative Commons license, unless indicated otherwise in a credit line to the material. If material is not included in the article's Creative Commons license and your intended use is not permitted by statutory regulation or exceeds the permitted use, you will need to obtain permission directly from the copyright holder.
To view a copy of this license, visit https://creativecommons.org/licenses/by/4.0/

<u>Response to Reviewers</u>

Manuscript ID: NCOMMS-26-015977

<u>Reviewer #1:</u>

The authors present a wave-based reservoir computing (RC) system in which the reservoir is a body of water. An array of actuators launches propagating surface waves in a container, where the actuators' trigger times are encoded from discrete sensory readings (in this work, obstacle distance). Different obstacle states in the test field therefore produce different wave-interference patterns, which provide a nonlinear transformation into a higher-dimensional representation space. The resulting wave-field images are then classified and linearly mapped to command classes for controlling a robotic rover. The authors also investigate how temporal and spatial dynamics affect recognition accuracy, and they outline a prospective path toward chip-scale implementations by simulating an analogous scheme using nanoscale spin waves. Overall, this is an interesting and potentially impactful contribution that could stimulate work in hardware-based AI, analog/edge computing, and neuromorphic computing and control. That said, several important questions should be addressed to clarify novelty, RC-specific functionality, and design choices:

Reply: We thank the reviewer for the positive assessment of our work and for noting its potential impact in AI hardware and edge computing. In the following, we respond to each of the reviewer's points and describe the changes made to the manuscript to address them.

R1-Q1. *A key advantage of reservoir computing is its ability to directly process spatiotemporal information in analog sensory streams. In the present work, however, the inputs are discrete distance readings that are first encoded into discrete actuator initiation times. Please clarify the intrinsic difference between this approach and previously reported spike-/event-encoded RC systems (e.g., Nature Electronics 4, 748–756 (2021)). In particular, what aspects of "wave-based" dynamics provide capabilities beyond a generic time-encoding front end followed by a dynamical medium?*

R1-A1. We agree with the reviewer that a key advantage of reservoir computing (RC) is its ability to process spatiotemporal information directly. In our work, the inputs are discrete distance readings and are therefore encoded into discrete trigger times for the actuators. We chose this encoding because it matches the sensing format used in the robotic task, not because the method is limited to discrete inputs. In principle, the same reservoir could also be driven by continuous analogue signals, provided that the transduction stage maps them reproducibly onto the physical input channels.

Our approach differs from spike- or event-encoded reservoir-computing systems in that the temporal encoding serves only as the input stage. The computation itself is performed by a spatially extended wave medium, where wave propagation, interference, and dispersion shape the reservoir state. As a result, information is processed not only in time but also across space, which is a distinct feature of the wave-based reservoir. Compared with a generic time-encoding front end followed by a dynamical medium, this provides a richer and more controllable mapping of the inputs into a high-dimensional feature space.

Considering the Reviewer's comment, and to make this point more explicit, we added the following text to the main manuscript (page 5, lines 131-134):

"The discrete timing map should be viewed as an input transduction step rather than the computation itself. The temporally evolving, spatially extended wave medium, where propagation, interference, and dispersion occur, mixes the encoded inputs into a high-dimensional spatiotemporal state and thus acts as the reservoir."

R1-Q2. *Another core characteristic of RC is that the reservoir state depends not only on the current input but also on recent input history (fading memory), enabling context- and trend-dependent outputs. How does the proposed water-wave reservoir implement and quantify this*

property? For example, can the authors report memory capacity (or an equivalent metric), demonstrate performance on a task that explicitly requires temporal integration, or otherwise show that decisions depend on prior inputs rather than solely on instantaneous distance-to-spike timing?

R1-A2. We thank the reviewer for raising this important point. In addition to the ability to process temporal signals highlighted by the reviewer, another key advantage of RC is its architectural simplicity, as it relies on the intrinsic dynamics of a single physical system rather than large networks of artificially engineered neurons and synapses. As stated in the abstract and explained in the Introduction, our main motivation for using RC in this work is this latter aspect, since a compact and fully functional neuromorphic hardware platform is required to demonstrate real-time robotic operation.

While the specific robotic task in this work does not explicitly require temporal memory, we agree with the reviewer that fading memory is important for tasks with temporal dependencies, such as sequential decision-making and time-series processing. The water-wave reservoir naturally exhibits fading memory through wave propagation, finite decay times, and boundary reflections. In fact, in our study of the temporal evolution of classification accuracy (Figure 3d and Supplementary Fig. 3), we observed oscillations in the accuracy. This behaviour is related to reflected waves reintroducing feature mixing. As a result, the system provides a form of short-term memory in which features from earlier inputs can merge with new inputs to support history-dependent pattern recognition. We have already discussed this point in the first paragraph of Supplementary Information S3. ("*Along with recognition accuracy peaked at ~150 ms, a secondary peak was observed near 1400 ms, and the comparison with the corresponding wave images (Fig. 3a in the main text) indicates that this arises from reflected waves. These reflections reintroduce distinctive features useful for recognition, suggesting that reflected waves act as a form of short-term memory, merging with new inputs to enable history-dependent pattern recognition. This is an essential capability for tasks such as time-series analysis and speech recognition, and it is promising for extending the wave-computing concepts developed here to other AI applications beyond robotics."*).

For both water-wave and spin-wave systems, short-term memory is expected to depend strongly on reservoir parameters such as its size, wavelength, excitation frequency, and operating regime (linear or nonlinear). Consequently, memory capacity is not just an intrinsic property of the wave medium itself but also depends on reservoir configuration. A meaningful optimization and quantification of memory capacity would therefore require a systematic exploration of a large multidimensional parameter space. In addition, standard memory-capacity evaluation requires the continuous processing of long sequential input streams, typically involving thousands of samples. Therefore, a comprehensive optimization and characterization of memory capacity would require a substantial long-term study. Furthermore, for spin-wave systems, performing a large number of MuMax simulations across such a broad parameter space would require substantial computational resources.

Although we observed features of short-term memory arising from reflected waves, we agree that this discussion was included only in the Supplementary Information and not in the original main text. Therefore, we have now added the following sentences to the main manuscript (page 7, lines 162–166), with a reference to the Supplementary Information for a more detailed discussion: *"We also observed a second peak near 1400 ms, which we attribute to the mixing of reflected waves and which serves as a signature of short-term memory in the system. This is promising for extending wave computing to a range of AI applications that require temporal data processing (see Supplementary Information S3 for a detailed discussion)."*

R1-Q3: *What is the basis for the chosen spatial arrangement of the six actuators? Please discuss whether there is an optimal actuator spacing (or layout) for maximizing separability/accuracy, and how sensitive performance is to this parameter. If possible, include a sweep (or an argument) relating spacing to wavelength, container dimensions, boundary conditions, and interference richness.*

R1-A3. The six-actuator layout was chosen on practical, rather than fully optimized, grounds. The actuators had to be placed along one side of the reservoir, while the opposite side had to remain open for optical illumination and imaging. Within these constraints, the actuators were arranged to (i) provide roughly uniform spatial coverage, (ii) ensure sufficient overlap of the propagating waves, and (iii) preserve a degree of symmetry, so that no single axis is preferentially encoded and artificially dominates the computation.

We agree that actuator spacing is an important control parameter. However, we did not test this experimentally, as it would require a larger study with multiple experimental setups, including different container sizes and actuator layouts, and is therefore beyond the scope of the present work. To address the reviewer's point, we have added the following text to Methods Section 1, which describes the water-wave setup (page 12, lines 315-319):

"The six-input layout was chosen under optical-access constraints rather than by a full geometric optimization. More generally, very small actuator spacing is expected to increase input correlation, whereas very large spacing reduces wave overlap and therefore weakens nonlinear mixing. The best performance should occur in an intermediate regime set jointly by wavelength, damping length, reservoir size and boundary reflections."

R1-Q4. *The spin-wave simulations suggest characteristic operating frequencies in the GHz range. How would a spin-wave implementation interface with or be useful for controlling systems whose relevant dynamics are orders of magnitude slower (e.g., robotics, environmental sensing)? Please discuss possible strategies (e.g., temporal multiplexing, buffering/windowing, event-driven sampling, or multi-timescale reservoirs).*

R1-A4: We agree that the intrinsic operating speed of the reservoir is much faster than both the robotic dynamics and the rate of incoming sensory data. However, a GHz spin-wave reservoir does not need to run continuously on the timescale of the robot. Instead, it can act as a fast physical co-processor that is queried whenever new sensory data arrive. This is similar to conventional robotic systems controlled by CMOS processors, where GHz electronics routinely process sensory data that change and are updated at much lower rates.

Additionally, as noted by the reviewer, faster RC operation offers several advantages. It provides room for time multiplexing, repeated evaluations, averaging over noisy readouts, buffering or windowing of incoming data, and multi-timescale processing, in which a fast reservoir handles local classification while slower logic performs higher-level control.

We agree that this point was not stated explicitly in the manuscript. We have therefore added the following paragraph to the main text (page 10, lines 265-270):

"For real-time robotic vehicle control, sensory data arrive at a much slower rate (kHz) than the operating speed of spin waves (GHz). Even though the system can simply act as a fast physical co-processor that is queried whenever a new sensory update arrives, the fast processing timescales leave room for repeated evaluations, time multiplexing, buffering or windowing, and multi-timescale control architectures. Therefore, this approach will be advantageous for future robotic applications that require complex input data processing."

R1-Q5. *The proposed reservoir relies on coherent wave dynamics. Is coherence necessary for the claimed RC functionality, or is it mainly one possible physical substrate? Prior memristive RC demonstrations do not exhibit prominent coherent wave behavior yet still achieve RC effects. Additionally, recent work (e.g., Sci. Adv. 11, eadu2663 (2025)) shows high state richness in electrically tuneable semiconductor grids without explicit wave coherence. Please clarify what unique neuromorphic/RC functions the present wave-based approach enables that are difficult to realize with those alternative reservoirs (e.g., specific advantages in controllable spatial modes, programmability, energy scaling, robustness, or task classes).*

R1-A5: We do not think that our reservoir depends on coherent wave dynamics, and we do not claim that coherence is required for RC. Coherence can help by producing well-defined

interference patterns and controllable spatial modes, which can increase the richness of the reservoir state. However, RC can also be realised in non-coherent systems, such as the memristive and semiconductor networks mentioned by the reviewer.

What matters most is not coherence itself, but the presence of correlations that allow the reservoir to map input information into distinguishable internal states. Simple coherent waves, such as plane waves, are not sufficient on their own, since they contain limited information beyond frequency, phase, and amplitude, and provide little memory. For wave-based reservoirs, scattering, mixing, and interference are needed to generate non-trivial dynamics. In the resulting wave patterns, correlations in the input, whether temporal, spatial, or spectral, can then be converted into useful features that support effective classification.

The advantages of the wave-based reservoir-computing approach are already discussed in the Introduction (page 3, lines 67-80) and the Conclusions section (page 11, lines 291-293) of the manuscript. To further address the reviewer's comment on comparisons with alternative reservoirs, we have now benchmarked spin-wave-based computing against other neuromorphic systems, including different reservoir-computing platforms, and included the results in Supplementary Information Section S8. Below, we summarise the key features that distinguish the wave-based RC approach from alternative reservoir platforms:

(i) **Simultaneous spatial and temporal information processing:** Unlike time-multiplexed reservoirs, which encode information only in temporal sequences, the distinct advantage of wave system is that it naturally distributes information across both spatial and temporal degrees of freedom. This creates a higher-dimensional feature space and stronger nonlinear mixing within a single compact physical layer, without the need for virtual nodes or delay lines.

(ii) **Post-fabrication programmability:** The nonlinear dynamics of the spin-wave reservoir can be continuously tuned through the external magnetic field or via spintronic methods and the excitation conditions. This allows reconfigurable operation without changing the device geometry. In many other physical RC systems, the reservoir response is largely fixed after fabrication.

(iii) **Intrinsic short-term memory:** Wave propagation, reflections, and finite decay times naturally generate fading memory and temporal echo states (see Supplementary Information S3 and Supplementary Fig. 3a). By contrast, many time-multiplexed RC systems require explicitly engineered feedback loops or delay lines to obtain similar memory effects.

(iv) **Scalability toward compact low-power hardware:** Spin-wave devices are compatible with CMOS-integrated magnetic-device architectures and do not rely on charge transport through the propagation medium. This offers a route toward compact and energy-efficient neuromorphic hardware.

All these points are now discussed in detail in Supplementary Information S8. In addition, we added the following statement to the Conclusion section of the main text (page 11, lines 287-290): "An indicative quantitative benchmarking of the spin-wave RC system against representative neuromorphic and physical RC platforms is provided in Supplementary Section S8 and S9 and Supplementary Table 1, highlighting its advantages in operating speed, energy, training simplicity, device fabrication, and hardware overhead."

Reviewer #2:

The paper by Zohar et al. presents a very interesting and potentially impactful case study of a physical reservoir computing system, implemented experimentally using water waves and explored via simulations using spin waves. While there is a substantial body of literature on physical reservoir computing and its computational capabilities, there are relatively few fully realized demonstrators—i.e., complete, built, and experimentally validated systems with all implementation details carefully addressed. In this regard, the present work is particularly valuable and timely. The authors demonstrate awareness of prior work on wave-based reservoir computing, and their hands-on, implementation-focused approach is highly appreciated. Overall, the study is well motivated and could provide an important contribution to the advancement of hardware-based reservoir computing.

Reply: We thank the reviewer for the encouraging and positive comments. As also noted in our response to R1-Q2, an important aspect of this work is that it is one of the few fully realised demonstrations of an autonomous system controlled by neuromorphic hardware. We present the system end to end, from building the experimental setup and validating its operation to real-time control of a robotic system using a fully functional neuromorphic platform. We greatly appreciate the reviewer's recognition of this distinct aspect of our work. Below, we address the specific questions raised by the reviewer.

R2-Q1. *However, despite these strengths, the manuscript currently lacks critical details and rigor required for publication in Nature Communications. There is no benchmarking against existing methods. The authors state that 100% accuracy can be achieved by selecting appropriate parameters, but this claim is presented without sufficient context or comparative evaluation. The chosen task—robotic navigation in a simple environment—is not particularly challenging, and the manuscript does not demonstrate the advantages of the proposed approach over conventional methods. The comparison is limited to a linear SVM, whereas more appropriate baselines, such as a simple multilayer perceptron (MLP) or convolutional neural network (CNN), would provide a fairer and more informative assessment. While the Supplementary videos are nice, they do not allow to quantify how difficult really the navigation task is.*

R2-A1. Our main achievement in this work is the proof-of-principle closed-loop obstacle-avoidance robotic task, demonstrating that wave-based RC has the potential to realize fully functional real-world wave-based RC hardware with simpler architectures compared with CMOS-based large spiking neural-network architectures. Performing more complex robotic tasks and other AI problem-solving tasks using different solid-state wave platforms is therefore a future perspective of this work. This has already been discussed in the Conclusions section of the paper. "Although our current demonstrations focused on basic robotic obstacle avoidance task, the same framework is readily extensible to more complex behaviours by increasing the input fan-in, combining reservoirs operating at different temporal and spatial scales, and incorporating multiple readout terminals"

However, we agree with the Reviewer that claims regarding future perspectives, benchmarking, and comparisons with other approaches should be supported more rigorously. We have therefore added two new sections (S8 and S9) to the Supplementary Information with detailed discussions.

In Section S9, directly addressing the Reviewer's comment, we added a comparison of SWRC with MLP and CNN architectures. For these comparisons, we did not train the MLP/CNN models on the robotic task itself, since this would not provide a meaningful computational comparison. The robotic task considered here was designed primarily to demonstrate the proof-of-principle closed-loop obstacle-avoidance operation of the physical wave-based RC system. To obtain a more meaningful comparison, we trained the networks on the 2D XOR classification benchmark task, which is a widely used benchmark for evaluating nonlinear computational capability. We used 2D images as input for all approaches, ensuring that all systems performed the same image-processing problem encountered in autonomous-vehicle

robotics. In this example, the SVM system used only two orders of magnitude less compute to achieve the same outcome as the MLP/CNN. Additionally, we have also calculated the kernel rank of our system, where the spin-wave RC system maintains a nearly full-rank, input-separable feature space across a broad range of spatial resolutions (See section S9.1)

Furthermore, Supplementary Information Section S8 also provides a comparison of wave-based RC approaches with other neuromorphic hardware platforms. As explained in R1-A5, the wave-based system shows several clear advantages in terms of simultaneous spatial and temporal information processing, CMOS compatibility, scalability, and architectural simplicity.

While adding these new comparisons in the two sections, we identified several important limitations that should also be acknowledged. First, many neuromorphic hardware systems are still an emerging technology, and many aspects of their ultimate performance limits and practical scalability remain uncertain. As such, direct comparisons between architectures should be interpreted with caution. In Section S8, we benchmark the performance metrics of the SWRC system, while for other neuromorphic architectures, we rely on information available in the literature and, in some cases, estimate future performance projections based on the reported device characteristics. Second, the performance of different reservoir hardware systems could depend on the specific computational task, reservoir design, input encoding strategy, and readout mechanism. Different neuromorphic platforms may therefore exhibit advantages for different classes of problems. For example, time-multiplexed RC systems may be advantageous for sequential temporal tasks, while wave-based RC systems naturally benefit from simultaneous spatial and temporal information processing through interference dynamics. In Section S9, we performed XOR task for generalized comparison, while there could be many other task comparison, which is beyond the scope of this work intending to demonstrate robotic task.

Considering these factors, we therefore emphasize in section S8, that "These benchmarking comparisons were performed based on the currently available information for the different technologies, which may evolve over time. Therefore, the comparisons presented in this section should not be interpreted as universal rankings between neuromorphic approaches, but rather as indicative comparisons for the specific task classes and implementation assumptions considered in this work".

Along with adding new Supplementary Information sections (S8 and S9), we added the following statement to the Conclusion section of the main text (page 11, lines 287-290): "An indicative quantitative benchmarking of the spin-wave RC system against representative neuromorphic and physical RC platforms is provided in Supplementary Section S8 and S9 and Supplementary Table 1, highlighting its advantages in operating speed, energy, training simplicity, device fabrication, and hardware overhead."

R2-Q2. *Furthermore, it remains unclear whether the proposed approach offers any practical benefit in terms of training efficiency or computational cost. The manuscript provides very limited detail on how the reservoir output is trained, and there is no discussion of the associated computational complexity or resource requirements. This omission makes it difficult to evaluate the claimed advantages of the system, in the context of online training for robotic navigation.*

R2-A2: The main advantage of RC is that only the output readout layer needs to be trained. This is the key reason why RC can reduce training cost compared with other neural-network approaches, where the internal layers also have to be trained, which is much more expensive in both computation and energy. This is the main motivation for our use of RC, as already stated in the Introduction (page 3, lines 61-63):

"In RC, only the output layers are trained (see Supplementary Information S1), significantly reducing the computing requirements and energy consumption."

We followed the standard RC training procedure, in which the features extracted from the reservoir output images were trained using a linear support vector machine (SVM). Because this is a low-cost computational task, it could be carried out on a CPU.

The reviewer raised a concern that the manuscript provides limited detail on how the reservoir output is trained. However, this procedure is already explained in detail in the Methods and in Supplementary Information S1. Specifically, the output image-processing pipeline is described in the Methods section, while the linear SVM training procedure is given in Supplementary Information Section S1. In addition, the training procedure is also mentioned in the main text (page 5, lines 128-131):

"The resulting waves propagated, interfered, and evolved dynamically, producing unique output interference patterns that were captured and used for training and testing with a support vector machine (SVM) (see Methods and Supplementary Information S1)."

We therefore believe that the output-training procedure is already described sufficiently. Nevertheless, if the reviewer feels that any key information is still missing, we would be happy to add it in the revised manuscript.

R2-Q3. *The least developed and most technically vague part of the manuscript is the simulation study involving spin waves. Essential details are missing: the geometry of the system is not described; it is not even clear what material is used in the ferromagnetic thin film. The cited GitHub repository lacks documentation and is poorly commented. Moreover, for spin-wave systems to function as reservoirs, they must operate in a weakly nonlinear regime. There is not a single word in the paper how this is achieved, what are the applied power levels at the input.*

R2-A3: The reviewer points out that essential details of the spin-wave simulations, including the system geometry, ferromagnetic thin-film parameters, and the linear and nonlinear properties of the spin waves, were missing. However, these points are already discussed in detail in Supplementary Sections S4, S5, and S6. We describe there that the simulations use a circular ferromagnetic disk with diameter of 1 μm and a thickness of 1.5 nm, with CoFeB-like parameters, M_{sat} = 1 MA/m and A_{ex} = 15 pJ/m (see Supplementary Information S5). We also address the reviewer's question about the weakly nonlinear operating regime and the input power levels. In the simulations, spin waves are excited via VCMA with a coefficient of 600-800 fJ/Vm, applied locally to each 50-nm transduction region (see Supplementary Information S5). This VCMA amplitude was chosen to place the reservoir in the weakly nonlinear regime: at low excitation amplitudes, the spin-wave dispersion is nearly linear, and the reservoir state maps almost directly onto the input, which provides insufficient feature diversity. At excessively high amplitudes, spin-wave turbulence leads to chaos and therefore irreproducible outputs. The operating point used here is intermediate, where the larger precession angle of the magnetization at finite amplitude introduces measurable nonlinear deviations from harmonic behaviour and generates the mode mixing required for RC.

We assume that the Reviewer's concern arises from the limited description in the main text. To address this, we have now added a concise summary of the spin-wave reservoir geometry and material parameters to the main text (page 10, lines 238-242):

"The spin-wave reservoir consists of a circular ferromagnetic CoFeB thin film with a diameter of 1 μm and a thickness of 1.5 nm, with a saturation magnetization (M_{sat}) = 1MA/m and an exchange stiffness (A_{ex}) 15 pJ/m. Using VCMA-driven excitation at six circular input transduction regions with a diameter of 50 nm"

R2-Q4: *Remarks on code availability: Minimal comments, no documentation for the python script running micromagnetic code. Choice of material parameters is unclear and some appears to be arbitrary. The T=8ns simulation time seems practically challenging to implement. No examples are given, no example data files, no links to the paper material The code is a useless resource at this point.*

R2-A4. We thank the reviewer and agree that the original repository was not documented well enough. We now have substantially expanded the code availability and documentation (https://github.com/jacobzohar/wave-based-neuromorphic-hardware). The updated GitHub repository now includes: (i) a comprehensive README with installation instructions, software requirements, and step-by-step procedures for reproducing the published figures; (ii) detailed documentation of the spin-wave geometry, material parameters, and simulation settings, together with their physical motivation; (iii) annotated MuMax3 source files and tutorial notebooks explaining the simulation workflow; (iv) a minimal end-to-end example demonstrating the complete classification pipeline from spin-wave simulation to machine-learning inference; and (v) bundled datasets and result caches that allow regeneration of all classification and benchmarking figures without rerunning the computationally intensive micromagnetic simulations. We have also added extensive inline comments, parameter descriptions, and documentation throughout the codebase to improve transparency and reproducibility.

In addition, we clarified the simulation parameters used in this work, including the material properties, excitation conditions, and simulation duration. The default simulation time used throughout the study is T_{max} = 4ns (6 ns for the coarse-step variant), which is now explicitly documented in the repository. Overall, the revised repository provides a fully documented and reproducible implementation of the spin-wave RC framework presented in this work.

<u>Reviewer #3:</u>

This work proposes a wave-based reservoir computing (RC) platform for robotic control, demonstrating its potential through water-wave experiments and spin-wave simulations. The concept of utilizing physical wave interference as a scalable and energy-efficient alternative to digital neuromorphic hardware is scientifically interesting. However, the manuscript suffers from technical overstatements and a lack of rigorous justification.

The term "navigation" is used loosely to describe what is essentially local obstacle avoidance, and the necessity of an RC framework for such low-complexity tasks remains unproven.

Furthermore, the claim of an "event-driven" mechanism lacks supporting technical details, and the transition to "practical" spin-wave chips is premature given the acknowledged experimental hurdles. Without quantitative benchmarking against state-of-the-art hardware and the resolution of logical inconsistencies, I cannot recommend the paper for publication in its current form. The following issues must be addressed:

Reply: We thank the reviewer for the careful review and for the positive comments on the scientific interest of our work. In the following, we address the reviewer's concerns about overstatements and the need for stronger justification, and we describe the corresponding revisions made to the manuscript.

R3-Q1. *The authors frequently use the term "robotic navigation" throughout the text and in the title. However, the actual functionality demonstrated by the system aligns more accurately with local "obstacle avoidance" or purposeless "wandering." In the robotics community, "navigation" strictly implies the capability to answer "Where am I?" and "Where am I going?"—typically involving localization, mapping, and global path planning toward a specific goal. The authors are strongly advised to revise the terminology across the manuscript and the title (e.g., replacing it with "obstacle avoidance" or "reactive control") to accurately reflect the scope of the work and prevent misleading the readers.*

R3-A1. We agree with the reviewer and have removed the term "navigation" from both the title and the main text. It has been replaced with "robotic reactive control," "robotic motion," "robotic operation," or, where appropriate, "robotic obstacle avoidance," depending on the context.

These changes are incorporated in the title, the abstract (lines 20 and 21), main text (lines, 39-40, 42, 43, 44, 83, 184, 185, 189, 191, 202, 203, 205, 224, 228, 278, 279, 282, 294, 345, 355, 375, and 377), and figure captions (Fig. 1 and Fig.4). We have also attached a version of the manuscript with all changes tracked for easy review.

R3-Q2. *There is an overstatement regarding the novelty of the experimental setup. The authors claim: "To overcome this, we first developed a unique experimental system based on water waves." However, as indicated by the authors' own reference [34] (Pattern recognition in a bucket), wave-based experimental setups for computation have been previously established. The authors should tone down absolute claims (e.g., removing "first developed") and objectively state the specific improvements or unique aspects their system offers over existing water-wave platforms.*

R3-A2. We agree with the reviewer. We would also like to add that, compared with previous systems, our setup incorporates several advances. We developed an Arduino-based control circuit that enables precise and stable control of the input wave excitations. This was essential for achieving robust operation, allowing long experiments with a large volume of input data and enabling reliable real-time control of the robotic system without high reliability. These aspects are described in the Methods and Supplementary Section S2.

To address the reviewer's concern, we have now removed the word "unique" to tone down the claim. The phrase "first developed" was not intended to imply priority, but only that this was the first step in our study. Nevertheless, we have removed the phrase and replaced it with "we built" (page 4, line 92). We also acknowledge that our system is similar to previously built

setups; however, it includes several advanced features, as clarified by adding the following text to the manuscript (page 5, lines 102-107):

"*This experimental setup has a configuration similar to the previously reported water-wave RC system[34]; however, it incorporates several advanced features, including an Arduino-based electronic circuit for temporally controlled and reproducible input generation on the ms timescale, a compact design that ensures strong wave interference, and placement on a vibration-isolated table to minimize external perturbations (see Methods and Supplementary Information S2)"*.

R3-Q3. *There is an inconsistency between Figure 2(f) and its corresponding caption. The text states "No object is the fifth class," but this description does not accurately correspond to the fifth condition visually presented in the figure. The authors should carefully check and revise the figure caption to ensure it perfectly matches the visual data, thereby avoiding reader confusion.*

R3-A3. We thank the reviewer for pointing out this error. In addition, the details of the class labelled "0" were explained only in the main text and were not sufficiently clarified in the figure caption. This class corresponds to two cases: either no obstacle is present, or the obstacle is sufficiently far away, beyond the defined threshold distance, such that the robot does not need to take any action to deviate from its path. We have now corrected this by revising the Figure 2 captions (d-f) as follows:

*"**(d)** 2-output task: Class 2 corresponds to an obstacle lying within the near field (minimum-distance range of 4), while Class 1 corresponds to either no obstacle being present within the sensor range or the obstacle being beyond the minimum-distance range. **(e)** 3-output task: Class 1 corresponds to either no object being present or the object being farther than a threshold distance (range 4). In the remaining two classes, an obstacle is detected within the threshold distance and is classified as being on either the left or the right side. **(f)** 5-output task: the first class corresponds to either no object being present or the object being beyond the far-distance threshold (range 4). The remaining classes correspond to obstacles grouped by distance, from "close" (range 2) to "far" (range 4), and then by lateral position (left or right)".*

R3-Q4. *For the specific application scenario presented in the study, the necessity of an RC system is insufficiently justified. If the task predominantly involves simple wandering or basic obstacle avoidance, such low-complexity objectives can typically be achieved using direct sensor feedback or simple threshold-based reactive control. The current experiments fail to demonstrate the unique advantages of RC. The authors should either introduce more complex tasks (e.g., processing complex spatio-temporal dynamics) or provide a robust discussion detailing why the RC system provides an irreplaceable advantage over traditional sensor-direct-control paradigms in this specific context.*

R3-A4. As mentioned in R1-A2, the choice of RC in this work is primarily motivated by its architectural simplicity. Machine-learning-based solutions require substantial computing resources and therefore motivate the development of emerging neuromorphic hardware solutions, where RC has a clear advantage because of its simpler architecture. However, as highlighted by Reviewer 2, there are still relatively few fully realised demonstrators of RC for applications such as robotic motion, where real-time neuromorphic control is required. The essence of this work is to take a first step in that direction.

However, we agree with the reviewer that the robotic task demonstrated here is relatively low in complexity, and that conventional robotics approaches based on machine learning can perform such tasks with fewer resources. Ideally, a more complex task should be used for a direct comparison. However, this would require processing a much larger number of parallel input injections, together with a corresponding increase in the number of input electrodes. Such studies are currently not feasible with a macroscopic water-wave system, while MuMax spin-wave simulations would require enormous computational time and resources.

Furthermore, real spin-wave device studies would require collaborative industrial efforts for the scalable optimization of nanoscale spintronic devices. Therefore, we currently present this as a future perspective of our work, as discussed in the Conclusions section.

Even though we cannot yet perform highly complex robotic tasks and directly compare them with conventional approaches, we agree with the reviewer that a more robust discussion is necessary. To address this, we have added several new discussions and analyses to the manuscript. The need for neuromorphic solutions, compared with conventional CNN-based approaches, is already discussed in paragraphs 2 and 3 of the Introduction. In addition, we now include benchmarking of the spin-wave system against other neuromorphic solutions in Supplementary Information Section S8 and S9, together with a detailed discussion. The details and limitations of these comparisons is provided in R1-A5 and R2-A1. In summary the wave-based RC system has several clear advantages compared with other RC systems. For example, CMOS spiking chips require custom fabrication processes, millions of transistors, dedicated on-chip learning circuits, and substantial peripheral infrastructure (Section S9, Supplementary Table 1). In contrast, the wave-based reservoir requires no weight-programming circuitry, consumes no dynamic power between input events, and can be fabricated from a single planar ferromagnetic film that is fully compatible with standard CMOS back-end processing. We also show that the present tasks can be solved using a relatively small number of output-MTJs (64 for the task in this work; see Supplementary Information Section S8). Taken together, these results indicate that future wave-based computing systems, with a larger number of input channels and a moderate number of output readout units, could perform much more complex tasks while potentially operating more efficiently than current conventional robotic solutions. In addition, we added the following statement to the Conclusion section of the main text (page 11, lines 287-290): “An indicative quantitative benchmarking of the spin-wave RC system against representative neuromorphic and physical RC platforms is provided in Supplementary Section S8 and S9 and Supplementary Table 1, highlighting its advantages in operating speed, energy, training simplicity, device fabrication, and hardware overhead.”.

R3-Q5. *There is a logical contradiction regarding the applicability of spin waves. The authors claim that spin waves can meet the requirements for "practical on-chip robotic systems." However, later in the text, they acknowledge that the "interference and readout of multiple nanoscale spin waves remain active experimental challenges" and rely purely on micromagnetic simulations for quantification. If the experimental readout and interference are yet to be realized, it is premature to claim the system meets "practical" requirements. The authors should soften this claim, presenting the spin-wave approach as a promising future direction rather than an already viable, practical solution.*

R3-A5. The SW RC system is compatible with chip-level device architectures, since the individual components of the proposed RC device, such as voltage gates and MTJs, are already integrated into CMOS fabrication lines (reference 48). At present, however, such integrations are primarily used for memory (MRAM) devices. Our study suggests that these technologies could also be used to realize practical SW RC devices. However, this will require further studies of spin-wave interference and readout in industrial-scale devices. By stating that “interference and readout of multiple nanoscale spin waves remain active experimental challenges,” we do not mean that these are impossible tasks. Rather, they remain relatively under-explored research directions, particularly at the industrial scale. Recognizing this, efforts to address these challenges are already emerging. For example, the study in reference 43 demonstrates the use of industrially fabricated MTJs for reading spin-wave information.

Addressing the reviewer’s comment, we have now changed the sentence (page 9, line 232) to:

“We therefore investigated SWs as a promising future chip-integrable platform for wave-based neuromorphic control.”

R3-Q6. *The manuscript mentions "sensory systems that trigger computation only upon dynamic changes in the environment." However, the technical details supporting this claim are missing. It is entirely unclear how this event-driven mechanism is physically or algorithmically realized using only a single transistor. The authors must provide a detailed explanation—and ideally a circuit logic diagram or operational principle schematic—to substantiate this claim.*

R3-A6. We thank and agree with the reviewer that this part is not explained in sufficient detail, and we have made the necessary modification in the main text.

The entire input excitation circuit of the physical RC system is powered by a 5 V power supply. We propose placing a switch between the circuit and the power supply; in a practical electronic implementation, a transistor switch would be the ideal choice, but it is not strictly required. To realise this scheme directly, the sensory circuit should be connected continuously to the wave-based RC system, with the switch placed in between. This would require both the sensory system and the RC system to be integrated onto the robotic car. In our current setup, however, the robotic car sensory system is connected wirelessly to the water-wave RC setup, making the direct placement of a physical transistor switch impossible. Therefore, as stated in the paper, this functionality was implemented virtually. In the Pygame environment, we introduced a transistor switch module on the car that turns on and off according to changes in the sensory input, indicated by the red and green colours in Supplementary Video 3. The RC computation is activated accordingly. At present, we use a virtual transistor switch, where the base voltage is activated by sudden changes in the sensory input, thereby turning on the transistor. We agree that, in the virtual implementation, this could in principle be any type of switch and not necessarily a transistor. However, in a real hardware implementation based on electronic circuitry, a transistor-based switch would be the most practical and appropriate choice.

The proposed circuit diagram is already shown in Fig. 4a. Agreeing with the reviewer that the operational details were not previously explained in sufficient detail, we have now made several modifications to the paragraph describing the event-based robotic operation. The revised paragraph (page 9, lines 205-225) now reads as follows:

*"*During its motion using the batch processing mode, the robot often encountered scenarios with no obstacles, leading to redundant computations of identical inputs. To mitigate this, we adopted an event-based control strategy in which neuromorphic processing is triggered only by significant changes in sensory input. In hardware, this can be implemented by converting distance-sensor signals to a baseline voltage that gates the wave-computing unit via a transistor switch (a proposed circuit is shown in Fig. 4a, bottom panel). However, to integrate the transistor switch practically, both the sensory unit and the RC system need to be integrated onto a chip mounted on the robotic car. In our current setup, the water-wave RC module is off-board and communicates wirelessly, making direct integration of transistor switch not feasible. We therefore implemented the event-based logic in the virtual robotic car (see Supplementary Video S3 and Fig. 4d). Here, we placed a virtual transistor switch between the sensor signal transmission unit and the RC system, indicated by a light turning red and green in Supplementary Video S3. In this mode, when there is no change in the sensory signal, the switch between the power supply and computing unit remains off, and the robot moves continuously without performing RC. Above-threshold changes in the sensory signal send a voltage to the transistor gate, turning on the transistor and thereby activating the RC system, and prompting the robot to make decisions according to the RC. The event mode of operation (Supplementary Video S3) reduced computational duty cycle resulting in faster overall operation and lower power consumption compared with the batch-processing mode (Supplementary video S2). Moreover, computation is triggered only when an event occurs, demonstrating the adaptability of event-based robotic operation to changes in the external environment.*"*

R3-Q7. *The authors propose wave-based computation as a "scalable and energy-efficient alternative." To make this claim scientifically convincing, qualitative descriptions are insufficient. The manuscript requires a detailed, quantitative comparison (e.g., in a tabular*

format) between the proposed wave-based system and representative state-of-the-art neuromorphic devices. Metrics such as energy consumption, computational efficiency, and hardware overhead must be explicitly compared to substantiate the claimed advantages of the proposed approach.

R3-A7. We agree that a quantitative comparison strengthens the scalability and efficiency claims, and we added a dedicated comparison table to the revised manuscript. As discussed in R1-A5, R2-A1, and R3-A4, we have added a new Section S9 and Supplementary Table 1 to the Supplementary Information, providing a detailed quantitative comparison across the following representative neuromorphic platforms: CMOS-based spiking chips (Intel Loihi, IBM TrueNorth), memristor crossbar arrays, spintronic nano-oscillator RC, photonic RC, and our spin-wave RC. The metrics compared include operating frequency, energy per operation, hardware overhead, device footprint, number of trainable parameters, training approach, CMOS compatibility, fabrication complexity, weight-reconfiguration requirements, and tasks demonstrated, directly addressing the metrics raised by the reviewer. The comparison indicates that the spin-wave RC offers GHz-speed operation, a nanoscale footprint, minimal hardware overhead, a single-layer training protocol, and CMOS process compatibility, supporting the scalability and energy-efficiency claims made in the manuscript.

As explained in R2-A1, we believe that such benchmarking relies partly on projections of the performance of different neuromorphic technologies, many of which are still at an early stage of development. Therefore, such comparisons should be interpreted with caution.

In addition to the new discussions added in Sections S8 and S9, we have also added the following statement to the main text (page 11, lines 287–290): *"*An indicative quantitative benchmarking of the spin-wave RC system against representative neuromorphic and physical RC platforms is provided in Supplementary Section S8 and S9 and Supplementary Table 1, highlighting its advantages in operating speed, energy, training simplicity, device fabrication, and hardware overhead.*"*

We believe this addition directly addresses the reviewer's concern and strengthens the manuscript's claims without overstating comparisons where the literature does not yet provide equivalent benchmarks.

Other Relevant Changes

Title: In response to Reviewer-2's comment, we have revised the title to "Autonomous robotic operation controlled by wave-based neuromorphic hardware."

Main Text: All newly added text and modifications are referenced in the corresponding reviewer responses. Compared with the original submission, we have repositioned all figures to improve page layout and formatting. These adjustments were necessary to accommodate the additional text introduced during the revision process, as detailed in the responses above.

Supplementary Information: We have added two new sections (Sections S8 and S9) that provide benchmarking results and computational-efficiency analyses. In addition, new references (Refs. 10-22) have been incorporated to support the expanded discussion.
